# A hybrid multi model artificial intelligence approach for glaucoma screening using fundus images

**DOI:** 10.1038/s41746-025-01473-w

**Published:** 2025-02-27

**Authors:** Parmanand Sharma, Naoki Takahashi, Takahiro Ninomiya, Masataka Sato, Takehiro Miya, Satoru Tsuda, Toru Nakazawa

**Affiliations:** Department of Ophthalmology, Tohoku University Graduate School of Medicine, Sendai, Japan

**Keywords:** Diagnosis, Medical research, Medical imaging

## Abstract

Glaucoma, a leading cause of blindness, requires accurate early detection. We present an AI-based Glaucoma Screening (AI-GS) network comprising six lightweight deep learning models (total size: 110 MB) that analyze fundus images to identify early structural signs such as optic disc cupping, hemorrhages, and nerve fiber layer defects. The segmentation of the optic cup and disc closely matches that of expert ophthalmologists. AI-GS achieved a sensitivity of 0.9352 (95% CI 0.9277–0.9435) at 95% specificity. In real-world testing, sensitivity dropped to 0.5652 (95% CI 0.5218–0.6058) at ~0.9376 specificity (95% CI 0.9174–0.9562) for the standalone binary glaucoma classification model, whereas the full AI-GS network maintained higher sensitivity (0.8053, 95% CI 0.7704–0.8382) with good specificity (0.9112, 95% CI 0.8887–0.9356). The sub-models in AI-GS, with enhanced capabilities in detecting early glaucoma-related structural changes, drive these improvements. With low computational demands and tunable detection parameters, AI-GS promises widespread glaucoma screening, portable device integration, and improved understanding of disease progression.

## Introduction

Glaucoma is an irreversible and progressive optic nerve disease, resulting in permanent loss of vision^1^. As life expectancy continues to increase, the number of glaucoma cases is on the rise with its prevalence surpassing that of conditions like diabetic retinopathy (DR) and age-related macular degeneration. This trend poses a significant public health challenge. Despite advancements in eye care, disparities in the availability of ophthalmologists, especially in resource-limited settings, highlight the need for scalable solutions^2^. Artificial intelligence (AI) and deep learning (DL) hold great promise in bridging this gap, particularly in automating the screening process for eye diseases.

AI technologies have already demonstrated significant success in automatic disease detection. Notably, the FDA-approved IDx-DR [Digital Diagnostics, Coralville, IA, USA, initially started as a project at the University of Iowa, under the name of the Iowa Detection Program (IDP)], and EyeArt AI systems have achieved significant milestones in diagnosing DR without requiring human interpretation^3,4^. These systems showcase AI’s transformative potential in eye care. However, glaucoma presents unique challenges that complicate the development of AI-based screening solutions^5^. Unlike DR, which relies primarily on fundus imaging, glaucoma diagnosis often requires a combination of information from fundus images, optical coherence tomography (OCT), intraocular pressure (IOP) measurements, and visual field (VF) testing^6–8^.

Glaucoma screening relies heavily on fundus imaging due to its cost-effectiveness, portability, and practicality in detecting glaucomatous features^9^. The screening process is conducted by analyzing the optic disc and surrounding areas [optic disc size and shape, cup to disc ratio (CDR), neuro-retinal rim, peripapillary atrophy, nasalization of blood vessels, presence of retinal nerve fiber layer defect (RNFLD), and optic disc hemorrhage (DH)]. Generally, a specialist can perform a qualitative assessment to detect glaucoma in less than 10 s. However, the initial stage known as pre-perimetric (PPG) is asymptomatic (minor/fewer changes in the retina). The optic nerve shows glaucomatous damage, but VF defects are not yet detectable by standard perimetric tests^10–14^. Therefore, sensitivity and specificity of VF testing in screening of early glaucoma is considerably low^15^. The PPG hinges on identifying minor structural changes in the retina^8^, which can challenge even experienced ophthalmologists. Factors such as DH and the extent of RNFL thinning are considered, but there is no consensus on a predictive model that accurately identifies progression risk for all patients, making PPG debatable in terms of diagnosis, progression, and treatment strategies. While technologies like OCT excel in detecting RNFLD, the identification of DH often requires fundus photography^16–20^. Nevertheless, detecting these early features during screening is crucial for identifying suspected glaucoma cases. Early detection enables timely intervention and close monitoring, thereby reducing the risk of blindness.

Real-world deployment of AI in glaucoma screening faces several hurdles, including variations in imaging modalities, differences in image quality, and population diversity^5,21^. While convolutional neural networks (CNNs)/DL models have shown excellent performance in binary classification (glaucoma/normal), their sensitivity to thresholds and overlapping image features limits their reliability, particularly for early-stage detection^22–31^. There are significant variations in reported results. For example, the area under the curve (AUC) varies from 71.8 to 99.7%, depending on the model used and the dataset’s ground truth definition. Typically, a binary classification model outputs a probability score between 0 and 1. The decision to classify a fundus image as either normal or glaucoma is based on the chosen threshold. When the features of glaucoma and normal images are well-separated, the model is likely to produce output probabilities that are confidently close to 0 or 1, leading to stable and good performance. However, the model’s performance becomes sensitive when there is significant overlap in image features between the two categories. As a result, the detection of moderate to advanced stages of glaucoma tends to be relatively accurate due to pronounced structural differences in fundus images. In contrast, performance becomes highly dependent on the chosen threshold, model architecture, and dataset in the early stages due to the subtle retinal changes^10–14^. Addressing these challenges requires advanced models capable of detecting minor retinal changes, which can be achieved by incorporating task-specific models.

Another challenge is the shortage of large, diverse clinical datasets for training AI models^9^. Variations in the characteristics of fundus cameras and image quality can significantly impact model performance. While transfer learning architectures like VGGNet, AlexNet, and GoogLeNet have been employed for image classification, these models are often optimized for non-clinical datasets (e.g., flowers or animals), limiting their effectiveness in glaucoma detection. Additionally, the high computational demands associated with deploying DL models pose obstacles to seamless integration with telehealth platforms. The lack of transparency in current AI models further complicates their adoption, as clinicians often require interpretable parameters to support decision-making. While a black-box approach can be highly effective for screening, an ideal scenario might involve a hybrid approach, where the algorithm not only provides predictions but also outputs critical parameters that assist in disease identification. This approach balances the strengths of AI-based screening with the need for transparency and trust in clinical practice.

Despite these challenges, macula-centered fundus images offer a promising pathway for glaucoma screening and the detection of other eye diseases beyond glaucoma^15,32^. These images provide a broader retinal view, enabling analysis of both the optic disc and macula. This perspective also facilitates the assessment of the RNFLD trajectory and provides the optic disc size index, which is essential for accurately interpreting the CDR. Although binary classification models trained on diverse fundus images can recognize disease-related features, they may overlook early indicators of glaucoma, such as RNFLD and DH, due to their emphasis on more prominent features like optic disc cupping. The automatic detection of RNFLD and DH^17,33^, by DL models remains challenging and is an underrepresented area in the existing literature.

Present studies have largely focused on estimating the optic disc parameters using segmentation models or predicting glaucoma through binary classification models. However, an integrated framework combining multiple diagnostic features is still lacking. To address this gap, we propose an AI-based glaucoma screening (AI-GS) network that acts as a virtual glaucoma specialist. Leveraging macula-centered fundus images, the AI-GS network predicts glaucoma while evaluating key optic disc features. By detecting multiple risk factors and integrating outputs from various models, this network enhances sensitivity to minor structural changes associated with glaucoma, significantly improving early detection during screening in comparison to binary classifiers. Moreover, the AI-GS network is designed to integrate seamlessly with telehealth platforms, advancing accessible and accurate glaucoma screening for timely intervention and prevention of vision loss.

## Results

### Architecture of AI-based glaucoma screening network

**Fig. 1:**
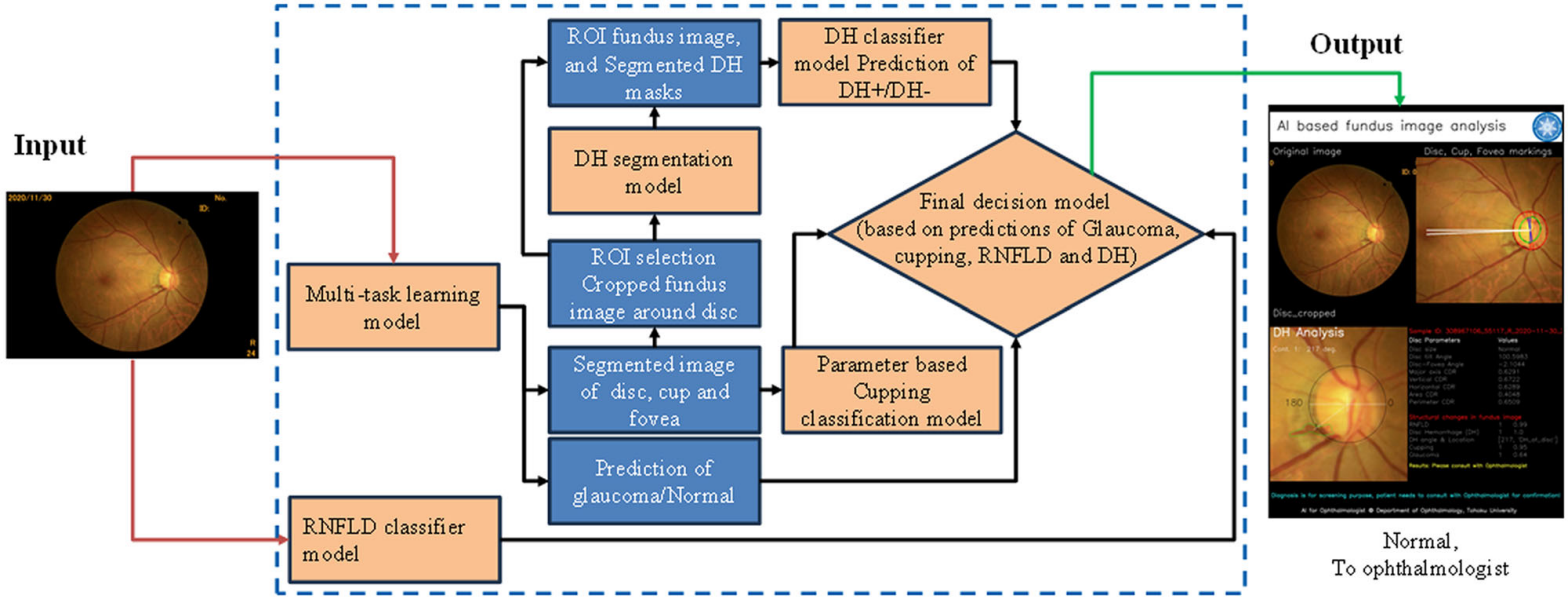
Architecture of the AI-based glaucoma screening (AI-GS) network. The network integrates multiple DL models to extract glaucomatous features from fundus images, such as disc hemorrhage (DH) and retinal nerve fiber layer defects (RNFLD), and measure key parameters like the cup-to-disc ratio (CDR) and disc size index. These parameters are combined through a fully connected network (FCN) to estimate glaucoma probability, which is further refined using image-wide predictions—incorporating direct DL outputs, RNFLD, and DH—to generate the final screening decision, along with corresponding numerical values.

Figure 1 shows the block diagram of the AI-GS network. It consists of multiple DL models (multitask learning, segmentation, and classification models). The input to the models is a square-shaped fundus image (512 × 512 pixels), processed with contrast limited adaptive histogram equalization (CLAHE) method. The input images for DH segmentation and classification models are cropped from the original fundus image (512 × 512 pixels) around the optic disc as a region of interest (ROI). The output includes an image report along with a CSV file. The image report depicts boundaries of cup and disc, position of the macula, and location of DH (if detected). It also includes the numerical values of disc assessment, detected structural changes, glaucoma decision based on direct DL output and cupping analysis along with the final recommendations. The detailed disc assessment parameters include disc size index, disc size (large, small), center of the disc, cup and fovea, disc fovea angle, CDR (major axis, vertical, horizontal, area, and perimeter), area of the neuroretinal rim, circularity index of the cup and disc, and the decision probabilities of different models. The AI-GS network includes six lightweight DL models for tasks such as segmentation, image classification, and decision-making based on numerical features or probabilities. The combined memory size of all the models is ~112 MB. The layered architecture of the DL models used in the AI-GS network is inspired from our previously reported lightweight DL model (LWBNA-unet)^34^. This model was specifically designed for biomedical image segmentation and has several advantages over the traditional Unet model, which is popular for this task. Compared to Unet, LWBNA-unet is significantly lighter, with its weight reduced by a factor of 10. It achieves better segmentation accuracy and demonstrates improved reproducibility during model training. This model was specifically used for segmenting DH in fundus photographs. Furthermore, the LWBNA-unet model’s versatility allows for easy modification to create a DL model for binary classification and multi-task learning.

### Binary classification model based on LWBNA-unet

**Fig. 2:**
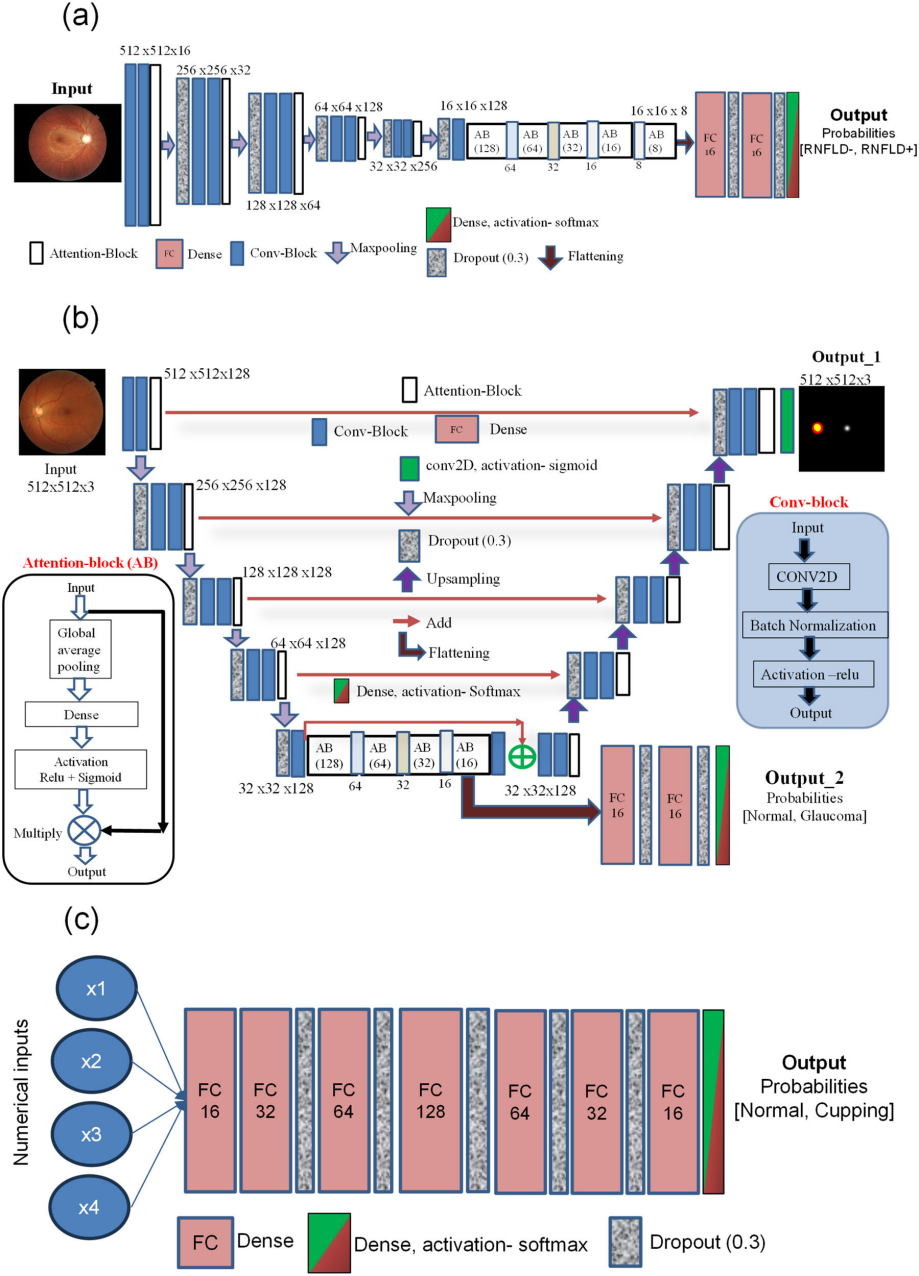
Architectures of lightweight models used in the AI-GS network. **a** Binary classifier based on LWBNA-unet model^34^, featuring the bottleneck narrowing with attention (BNA) module, which reduces the model’s parameter count without compromising prediction accuracy. **b** Layer details of the multi-task deep learning model based on LWBNA-unet (MTL_LWBNA-unet). **c** Feedforward fully connected network (FFCN), which combines numerical values and probabilities from the classification models to produce a binary output.

The architecture of the binary classifier used for identifying fundus images with the RNFLD and DH is shown in Fig. 2a. The encoder part and the Bottleneck Narrowing with Attention (BNA) module, i.e., the midblock, of the LWBNA-unet, were used^34^. The encoder takes a three-channel RGB image as input. In the first convolutional layer, the input image is expanded into a 16-channel feature map using 16 different filters. These filters detect low-level image features such as edges, textures, and patterns. In successive layers of the encoder, the number of channels (or filters) doubles, while the image size is halved. This enables the model to learn more complex and abstract features. At the end of the encoder, the initial matrix, which was 512 × 512 x 16 (width x height x channels) in the first layer, transforms into a feature matrix of size 16 × 16 x 256. The mid-block, or BNA module, further refines the extracted features and reduces the dimensions to the smallest size (16 × 16 x 8). The BNA module applies channel narrowing with attention, a technique that selectively focuses on the most relevant channels while discarding less informative ones. This process condenses the high-level image features into a more compact representation. The resulting multi-dimensional array (16 x 16 x 8) is then converted into a single-dimensional vector by flattening. Flattening stacks the values in the array row-by-row, creating a long continuous vector that retains the condensed high-level features in a linear format. Before reaching the binary output layer (the final layer that produces the classification prediction), the flattened vector is connected to fully connected (FC) and dropout layers twice. The FC layers help in learning complex relationships between the features, and dropout layers reduce overfitting by randomly disabling some neurons during training. The total number of model parameters is 1,718,770, which represents the weights and biases learned during the training process. Despite the large input image size, the model is relatively small at approximately 20.5 MB, achieved through channel narrowing with attention. This technique reduces the number of parameters while maintaining prediction accuracy, making the model more efficient and lighter.

### Multi-task learning model based on LWBNA-unet

Segmentation and classification tasks are crucial in medical image analysis, and certain scenarios require the achievement of both objectives. For instance, DL can directly classify fundus images as glaucoma or normal. Traditionally, the CDR has been employed for this purpose, necessitating the segmentation of cup and disc regions in the fundus image. The LWBNA-unet offers an efficient and flexible solution. It can be tailored for multi-tasking, performing both segmentation and classification within a single model. Multi-tasking enhances computational efficiency, reduces model size, and enables end-to-end learning. Figure 2b shows the details of the layers in the LWBNA-unet-based multi-task learning model (MTL_LWBNA-unet). The segmentation output is a three-channel image corresponding to the disc (red), cup (green), and fovea (white). A new branch from the end of the bottleneck between the encoder and decoder paths of LWBNA-unet is created to output classification of image into glaucoma/normal categories. Like the classification model (Fig. 2a), the multi-dimensional array size is smallest (32 × 32 x 16) at this branching point. It is then converted to a single dimension vector by flattening and followed by FC and dropout layers twice before the binary classification output layer (Fig. 2b).

### Model for numerical parameter-based classification

The AI-GS network also incorporates a DL model to make decisions using numerical values and probabilities derived from classification and segmentation models. For example, disc assessment parameters—such as vertical and horizontal CDR, disc size index, and cup and disc circularity index—determined from the segmented images of the cup, disc, and fovea are used to classify the fundus image as glaucoma or normal, i.e., a cupping-based decision. For this purpose, we used a simple feedforward FFCN (Fig. 2c). The model consists of five dense layers, each followed by a dropout layer for regularization. The number of neurons in each layer increases from 16 to 32, 64, 128, and then decreases to 64, 32, and 16. The final dense layer, which contains binary classification category neurons, uses the softmax activation function for multi-class classification.

### Segmentation of cup, disc and fovea by LWBNA-unet

**Fig. 3:**
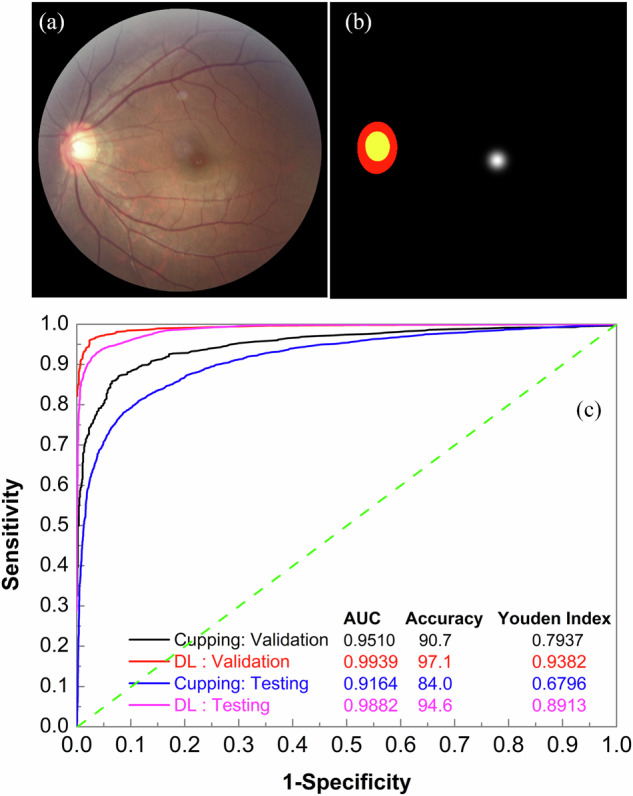
Example of input images and glaucoma screening results from binary classification models. **a** Macula centered fundus image. **b** Corresponding mask showing the optic disc (red + yellow), cup (yellow), and fovea (white) used for training the of MTL_LWBNA-unet model. **c** ROC curves comparing the best-performing MTL_LWBNA-unet and the cupping parameter based FFCN model. The MTL_LWBNA-unet model demonstrates superior glaucoma prediction performance compared to the cupping parameter-based approach.

Figure 3a, b shows an example of a fundus image and its mask created using the Eq. (1) for the REFUGE Dataset (as described in the “Methods” section). The disc is marked with red, the cup in yellow, and the fovea in white. The average D obtained on the validation dataset for disc and cup after six rounds of training with the LWBNA-unet is 0.9582 ± 0.0204 and 0.8896 ± 0.0573, respectively. The best values of D for disc and cup are 0.9601 ± 0.0202 and 0.8895 ± 0.0609, respectively. The performance of the LWBNA-unet is very close to the best results reported for this dataset^35^. As described in the “Datasets” section, an extended dataset was created using Topcon’s fundus images using the trained LWBNA-unet model. Remarkably, the D for the cup, disc, and fovea were consistent with those obtained without incorporating the Topcon fundus images, differing only at the third and fourth decimal places. These outcomes indicate the robustness of the model for cup, disc, and fovea segmentation, making it suitable for generating masks for diverse fundus images.

### Glaucoma prediction and estimation of cupping parameters by MTL_LWBNA-unet

Training of the MTL_LWBNA-unet model utilized 2,953 fundus images of glaucoma and 5,195 normal images, gathered from various sources (as described in the “Dataset” section)^36–38^. The masks for the cup, disc, and fovea (Fig. 3a, b), corresponding to each fundus image, were generated by the trained LWBNA-unet model as described earlier. Five-fold cross-validation of the MTL_LWBNA-unet model was conducted. After training five times, ROC curves were plotted for each validation dataset. The average values along with the standard deviation (std) for the five-fold cross-validation testing at a threshold of 0.5 are as follows: prediction accuracy = 96.2812 ± 0.4451%, AUC = 0.9904 ± 0.0014, specificity = 0.9692 ± 0.0074 and sensitivity = 0.9516 ± 0.0151. The low std values suggest that the model’s performance is consistent, and likely to generalize well to unseen images. Figure 3c shows the ROC curve of the best-performing model among the five trained models. At an optimum threshold of 0.5245, sensitivity and specificity for the detection of glaucoma on the validation dataset by DL is ~ 96.19% and ~ 97.63%, respectively. Details of the results, along with the 95% CI, are given in Table 1. These results highlight the model’s effectiveness in detection of glaucoma, demonstrating its potential for clinical application.

**Table 1:** Glaucoma detection results for the validation, testing, and real-world screening datasets, using different DL models for classification and segmentation based on LWBNA

Models/Network	Datasets	AUC	Accuracy	Sensitivity	Specificity	Youden Index
Cupping parameter based FFCN	Training validation	0.9510	0.9070	0.8599	0.9339	0.7937
	95% CI (LL, UL)	(0.9389, 0.9629)	(0.8931, 0.9214)	(0.8316, 0.8875)	(0.9174, 0.95)	(0.7621, 0.8253)
	Testing	0.9164	0.8398	0.7448	0.9348	0.6796
	95% CI (LL, UL)	(0.9103, 0.9225)	(0.832, 0.8477)	(0.7316, 0.7576)	(0.9274, 0.9425)	(0.6644, 0.6951)
	PPG	0.8503	0.7259	0.5069	0.9448	0.4517
	95% CI (LL, UL)	(0.819, 0.8782)	(0.6897, 0.7603)	(0.4485, 0.5619)	(0.9199, 0.9701)	(0.3879, 0.5104)
	POAG	0.9333	0.8609	0.7749	0.9468	0.7218
	95% CI (LL, UL)	(0.9236, 0.9423)	(0.8481, 0.8733)	(0.753, 0.7968)	(0.9353, 0.9598)	(0.6963, 0.7458)
	NTG	0.9196	0.8456	0.7557	0.9354	0.6911
	95% CI (LL, UL)	(0.9119, 0.9276)	(0.8354, 0.8547)	(0.739, 0.7718)	(0.925, 0.9449)	(0.6713, 0.7089)
	Miyagi Screening	0.9247	0.8403	0.9244	0.7561	0.6805
	95% CI (LL, UL)	(0.9079, 0.9388)	(0.8176, 0.8611)	(0.9008, 0.9457)	(0.7197, 0.7894)	(0.6381, 0.7192)
MTL_LWBNA-unet	Training validation	0.9939	0.9711	0.9619	0.9763	0.9382
	95% CI (LL, UL)	(0.9906, 0.9965)	(0.9629, 0.9793)	(0.9457, 0.977)	(0.9664, 0.9853)	(0.9205, 0.9554)
	Testing	0.9882	0.9456	0.9314	0.9599	0.8913
	95% CI (LL, UL)	(0.9866, 0.9898)	(0.9409, 0.9503)	(0.9234, 0.9384)	(0.9543, 0.9662)	(0.8819, 0.9008)
	PPG	0.9707	0.8845	0.8069	0.9621	0.7690
	95% CI (LL, UL)	(0.9591, 0.9805)	(0.8586, 0.9069)	(0.7636, 0.8516)	(0.9395, 0.9818)	(0.7179, 0.8141)
	POAG	0.9896	0.9479	0.9315	0.9643	0.8958
	95% CI (LL, UL)	(0.9871, 0.9921)	(0.9385, 0.9563)	(0.9169, 0.9446)	(0.9527, 0.974)	(0.8779, 0.9129)
	NTG	0.9909	0.9532	0.9457	0.9607	0.9064
	95% CI (LL, UL)	(0.9891, 0.9926)	(0.9473, 0.959)	(0.9361, 0.9545)	(0.9533, 0.9679)	(0.8945, 0.918)
	Miyagi Screening	0.8783	0.7514	0.5652	0.9376	0.5028
	95% CI (LL, UL)	(0.8571, 0.8992)	(0.724, 0.7769)	(0.5218, 0.6058)	(0.9174, 0.9562)	(0.4556, 0.5499)
DH Classification	Training validation	0.8394	0.7856	0.7512	0.8135	0.5647
	95% CI (LL, UL)	(0.8025, 0.8771)	(0.7484, 0.8206)	(0.6952, 0.8113)	(0.7656, 0.8571)	(0.4881, 0.6395)
	Testing	0.8723	0.7549	0.5889	0.9218	0.5107
	95% CI (LL, UL)	(0.8362, 0.9042)	(0.7131, 0.7967)	(0.5116, 0.6609)	(0.88, 0.9588)	(0.4323, 0.5891)
DH Mask Assisted Classification	Training validation	0.9591	0.8972	0.9268	0.8730	0.7998
	95% CI (LL, UL)	(0.942, 0.9732)	(0.8687, 0.9234)	(0.8877, 0.9592)	(0.8287, 0.9116)	(0.7464, 0.8545)
	Testing	0.9678	0.9053	0.8722	0.9385	0.8108
	95% CI (LL, UL)	(0.9507, 0.9814)	(0.8746, 0.9331)	(0.8222, 0.9209)	(0.9006, 0.9701)	(0.7472, 0.8667)
RNFLD Classification	Training validation	0.993272557	0.959677419	0.957198444	0.960985626	0.91818407
	95% CI (LL, UL)	(0.9893, 0.9967)	(0.9462, 0.9731)	(0.9294, 0.9804)	(0.9436, 0.9768)	(0.8871, 0.9468)
AI-GS	Testing	0.9567	0.9370	0.9388	0.9352	0.8741
	95% CI (LL, UL)	(0.9517, 0.9611)	(0.9315, 0.9419)	(0.9316, 0.9459)	(0.9267, 0.9424)	(0.8631, 0.884)
	PPG	0.9123	0.8879	0.8310	0.9448	0.7759
	95% CI (LL, UL)	(0.8873, 0.9371)	(0.8603, 0.9138)	(0.7864, 0.8738)	(0.9181, 0.9682)	(0.7217, 0.825)
	POAG	0.9591	0.9432	0.9359	0.9505	0.8864
	95% CI (LL, UL)	(0.9505, 0.9672)	(0.9341, 0.9512)	(0.9231, 0.9482)	(0.9391, 0.9608)	(0.8682, 0.9024)
	NTG	0.9630	0.9467	0.9528	0.9405	0.8933
	95% CI (LL, UL)	(0.9571, 0.9686)	(0.9407, 0.9526)	(0.9446, 0.9614)	(0.9316, 0.95)	(0.8812, 0.9053)
	Miyagi Screening	0.9253	0.8582	0.8053	0.9112	0.7164
	95% CI (LL, UL)	(0.9099, 0.9406)	(0.8374, 0.879)	(0.7704, 0.8382)	(0.8887, 0.9356)	(0.675, 0.7577)
AI-GS Adjusted	Testing	0.9567	0.9127	0.9575	0.8679	0.8253
	95% CI (LL, UL)	(0.9519, 0.9615)	(0.9069, 0.9185)	(0.9513, 0.9636)	(0.858, 0.8765)	(0.8141, 0.8371)
	PPG	0.9123	0.8862	0.8759	0.8966	0.7724
	95% CI (LL, UL)	(0.8842, 0.9354)	(0.8586, 0.9104)	(0.8357, 0.9119)	(0.86, 0.9306)	(0.7192, 0.8231)
	POAG	0.9591	0.9275	0.9578	0.8973	0.8551
	95% CI (LL, UL)	(0.9512, 0.9666)	(0.9184, 0.9366)	(0.9466, 0.9677)	(0.8812, 0.9125)	(0.836, 0.8735)
	NTG	0.9630	0.9217	0.9667	0.8767	0.8434
	95% CI (LL, UL)	(0.957, 0.9684)	(0.9144, 0.929)	(0.9588, 0.9736)	(0.8641, 0.8896)	(0.8289, 0.8578)
	Miyagi Screening	0.9253	0.8752	0.9301	0.8204	0.7505
	95% CI (LL, UL)	(0.9094, 0.941)	(0.8554, 0.8941)	(0.9089, 0.9506)	(0.7862, 0.8512)	(0.7114, 0.7882)

*p* values for all AUCs are extremely small (effectively close to 0), indicating high statistical significance.

*CI* confidence intervals, *LL* lower limit of 95% CI, *UL* upper limit of 95% CI, *AUC* area under the receiver-operator characteristics curve, *AI-GS* AI-based glaucoma screening, *MTL_LWBNA-unet* multi-task learning light weight bottleneck narrowing with attention in Unet architecture, *DH* disc hemorrhage, *RNFLD* retinal nerve fiber layer defects, *PPG* pre-perimetric glaucoma, *NTG* normal tension glaucoma, *POAG* primary open-angle glaucoma.

Traditionally, glaucoma diagnosis relies on analyzing optic disc and cup features in fundus images. The present MTL_LWBNA-unet model segments the optic disc, cup, and fovea boundaries, enabling the estimation of size and shape for various fundus image features. We utilized several parameters for glaucoma detection, including the disc size index (disc-to-fovea distance/maximum disc diameter), major axis CDR, vertical CDR (VCDR), horizontal CDR (HCDR), area CDR (ACDR), perimeter CDR (PCDR), neuro-retinal rim area (NRRA)/disc area (DA) ratio, and circularity indices (CI) for both the disc and cup. These parameters were used to train a simple FFCN model, as shown in Fig. 2c. The ROC curve of the best-trained FFCN model from five-fold cross-validation is shown in Fig. 3c. The results obtained at the optimal threshold of 0.4638 are presented in Table 1. As anticipated, glaucoma detection using cupping parameters (sensitivity = 85.99% and specificity = 93.39%) is less effective than the DL-based approach.

**Fig. 4:**
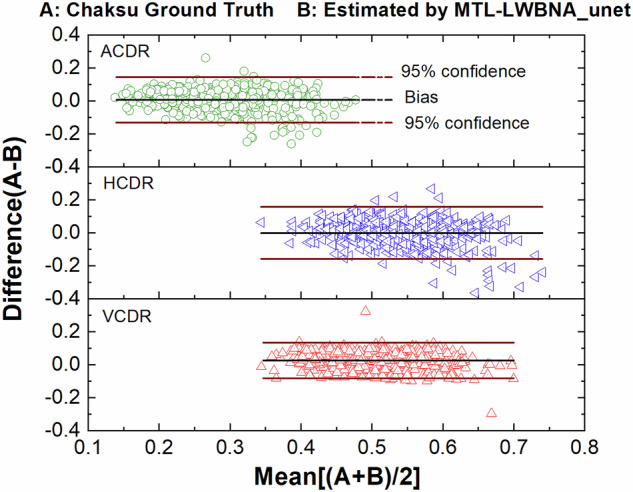
Comparison of optic disc cupping parameters estimated by the MTL_LWBNA-unet model with those manually estimated by experts. Bland-Altman plots comparing the vertical cup-to-disc ratio (VCDR), horizontal cup-to-disc ratio (HCDR), and area cup-to-disc ratio (ACDR) automatically estimated by the deep learning model with expert-derived values from the Chaksu dataset. In each plot, the centerline represents the bias, while the upper and lower lines indicate the limits of agreement (mean difference ± 1.96 times the standard deviation of differences). Values near zero indicate close agreement between the MTL_LWBNA-unet model’s estimates and the experts’ manual estimates.

To assess the precision of the MTL_LWBNA-unet model in segmentation and CDR estimation, we used a distinct dataset known as “Chaksu”^39^. Out of 500 images, we successfully calculated the VCDR, HCDR, and ACDR for 481 images (85 from Bosch, and 396 from Remidio cameras). We compared these results with data annotated by five experts in the Chaksu dataset. The dataset provides mean, median, and the majority values for VCDR, HCDR, and ACDR as assessed by different experts. To compare the accuracy of automatic CDR estimation by MTL_LWBNA-unet with manual estimation by experts, we conducted Bland-Altman analysis—an approach that assesses agreement between two quantitative measurements. Figure 4 illustrates Bland-Altman plots comparing VCDR, HCDR, and ACDR estimated from the present model and majority values from Chaksu dataset experts. In each plot, the centerline represents bias, while the upper and lower lines depict limits of agreement (mean difference ± 1.96 times the std of differences). This range reflects the expected variability between the two methods (i.e., ~95% CI). The bias and std of differences in VCDR, HCDR and ACDR are 0.0256 ± 0.0552, −0.0020 ± 0.0806, and 0.0057 ± 0.0701, respectively. These values, approaching zero, indicate close agreement between MTL_LWBNA-unet estimates and manually estimated values from experts. To understand further the variability in measurements, we plotted the Bland-Altman plots of five experts with majority values in the Chaksu dataset, and the results are given in Table 2. Comparison of DL-based parameters with experts suggests that the current model outperforms some experts, with a higher percentage of data points falling within the 95% CI.

**Table 2:** Results of Bland-Altman plots of five experts with majority values in the Chaksu dataset

	VCDR			HCDR			ACDR		
	Bias	std	% of data in 95% confidence	Bias	std	% of data in 95% confidence	Bias	std	% of data in 95% confidence
Majority vs. Expert_1	0.0081	0.0519	94.59	0.0191	0.0527	95.43	0.0105	0.0397	94.59
Majority vs. Expert_2	−0.0385	0.0674	93.97	−0.0291	0.0703	92.93	−0.0442	0.0723	95.63
Majority vs. Expert_3	0.0018	0.0587	97.09	−0.0061	0.0570	96.67	−0.0053	0.0613	97.92
Majority vs. Expert_4	−0.0125	0.0488	94.80	−0.0344	0.0525	94.59	−0.0279	0.0457	94.59
Majority vs. Expert_5	0.0232	0.0453	93.97	0.0632	0.0743	96.05	0.0411	0.0498	95.22
Majority vs. MTL_LWBNA-unet	0.0256	0.0552	96.47	−0.0020	0.0806	96.05	0.0057	0.0701	96.26

*VCDR* vertical cup to disc ratio, *HCDR* horizontal cup to disc ratio, *ACDR* area ratio of cup to disc, *Std* standard deviation, *MTL_LWBNA-unet* multi-task learning light weight bottleneck narrowing with attention in Unet architecture.

To test the classification accuracy of glaucoma and non-glaucoma cases, we created a balanced testing dataset of 8370 images. None of the fundus images in the testing dataset were part of MTL_LWBNA-unet training or validation datasets. Figure 3c shows the ROC curves illustrating glaucoma classification via direct model prediction and based on parameters estimated for cupping. The testing results are summarized in Table 1, highlighting that direct predictions by the MTL_LWBNA-unet significantly outperformed parameter-based predictions. Specifically, parameter-based predictions achieved a sensitivity of 70.8% at 95% specificity, while the DL approach attained 94.0% sensitivity, significantly exceeding the 85% sensitivity threshold recommended by Prevent Blindness America for glaucoma screening tools^40^. As noted in the introduction, a primary goal of this study is to detect PPG/suspected glaucoma cases. To achieve this, we segregated PPG, NTG, and POAG cases from the testing dataset to create specialized subsets. Glaucoma detection results are presented in Table 1. Predictions for NTG and POAG groups were comparable, while performance for the PPG group was notably lower. Performance was poorest in real-world scenarios like the Miyagi Screening, with sensitivities at 95% specificity as follows: 48.9% (Miyagi Screening), 83% (PPG), 94.2% (POAG), and 95.3% (NTG). A similar trend was observed in parameter-based predictions (Table 1). The markedly lower cupping-based sensitivity for PPG ( ~ 47% at 95% specificity) reflects minimal optic disc changes in early glaucoma stages. This highlights the importance of detecting subtle structural changes for accurate PPG detection. The model’s superior direct prediction accuracy likely stems from its ability to detect glaucomatous features beyond optic disc changes. Interestingly, the cupping-based sensitivity for Miyagi Screening at 95% specificity (~60%) exceeded that of the DL model. This suggests that ophthalmologists’ screening decisions may heavily rely on optic nerve head cupping along with other structural changes. Integrating specialized DL models that detect structural changes such as DH and RNFLD into a network could potentially enhance the performance of screening PPG and glaucoma in real-world scenarios.

### Detection of disc hemorrhage

**Fig. 5:**
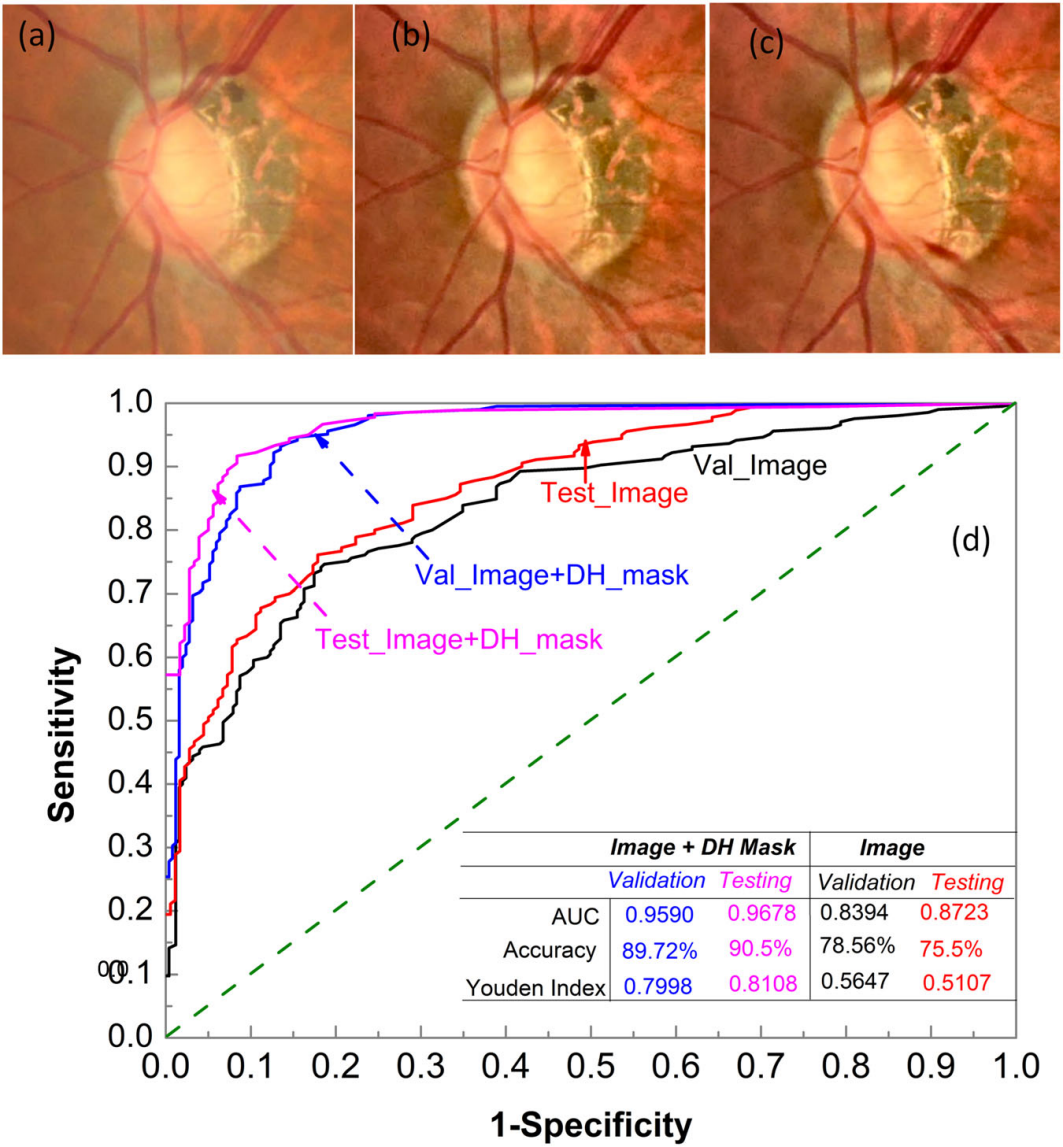
Automatic detection of disc hemorrhage (DH) in a fundus image. **a** Typical example of a DH-negative image cropped around the optic disc. **b** Intensity-equalized image using the CLAHE algorithm. **c** Appearance of DH at the inferior-temporal boundary of the optic disc, highlighting DH as a time-dependent phenomenon. **d** ROC curves for the validation and testing datasets. The results indicate that DH detection accuracy in binary classification improves significantly when the probable DH area is provided through segmentation, i.e., using a DH mask.

Detecting DH is crucial for accurate glaucoma diagnosis. DH appears as circular or flame-shaped patches of bleeding around the optic disc. Patients with DHs face a higher risk of glaucoma progression, requiring closer monitoring and timely intervention. DHs have been identified as an independent risk factor for the conversion of ocular hypertension to POAG or the progression of open-angle glaucoma^17,18,41^. Automatically detecting DHs from fundus photographs remains challenging, as they have been resistant to DL-based detection. Notably, publicly available datasets for DH remain scarce. To address this, we created a dataset for training LWBNA-based binary classification model. Representative image examples are shown in Fig. 5a–c. The model input is a 512 × 512 pixels image cropped around the optic disc. We used the original image (Fig. 5a) and its intensity-equalized version, processed using the CLAHE algorithm (Fig. 5b). Binary classification model (Fig. 2a) was trained ten times, and the best-performing model was selected based on validation accuracy. Figure 5d shows ROC curves for both the validation and testing datasets, with a summary of results in Table 1. On the validation dataset, the top-performing binary DH classification model achieved an AUC of 0.8394, an accuracy of 78.56%, and a Youden index of 0.5647 at a threshold of 0.5093. On the more balanced testing dataset, consisting of single image per eye, the model achieved an AUC of 0.8723, an accuracy of 75.49% and a Youden index of 0.5879 at a threshold of 0.4373. The predictive accuracy of the DH classification model is notably lower than that of the glaucoma classification model. This discrepancy may be explained by the small size of DHs and their similarity in color to other retinal features, such as vessels and pigmentation.

**Fig. 6:**
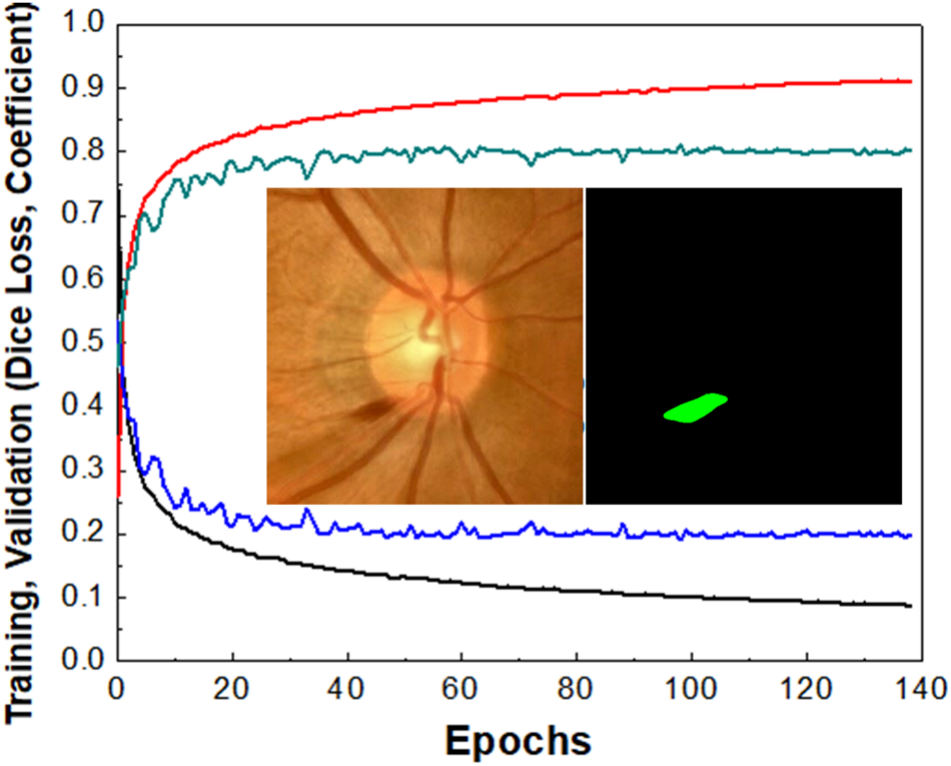
Segmentation of the disc hemorrhage region in a fundus image. Training and validation curves of the LWBNA-unet model trained for DH segmentation. The inset shows a typical example of an input image and its corresponding DH mask. A high Dice coefficient indicates successful delineation of the DH area by the segmentation model.

To enhance the classification accuracy of DH, we can provide the algorithm with spatial information about possible DH location. This approach mimics an ophthalmologist’s process of searching for DH in fundus images. For instance, initial observations may focus on retinal pigmentation or identifying true DH + , especially when they are large. Additionally, localized vessel contrast may be considered. However, a careful examination of these locations can help in ruling out false DH+ cases. To provide spatial information to the algorithm, we can either create masks outlining possible DH+ areas through segmentation. Alternatively, we can use bounding boxes, similar to object localization in object detection models. Although segmentation requires more effort to generate masks, it provides a detailed characterization of DH, including size, precise location, shape, and other attributes. Therefore, we created a dataset of 1704 DH+ fundus images and their corresponding masks. The LWBNA-unet segmentation model was trained using dice loss, similar to the approach used for segmenting FAZ in OCTA images^34^. A typical example of the image and its corresponding mask input to the model is shown in the inset of Fig. 6. Despite the often-unclear boundaries of DH, the model’s training and validation curves are consistent and repeatable (Fig. 6). We achieved a significantly high D of 0.8291 ± 0.1576 for the validation dataset (296 images). In many cases, the DH segmentation produced by our model surpasses the precision of the ground truth masks used for training. This improvement is illustrated in Supplementary Figs. 1 and 2.

**Fig. 7:**
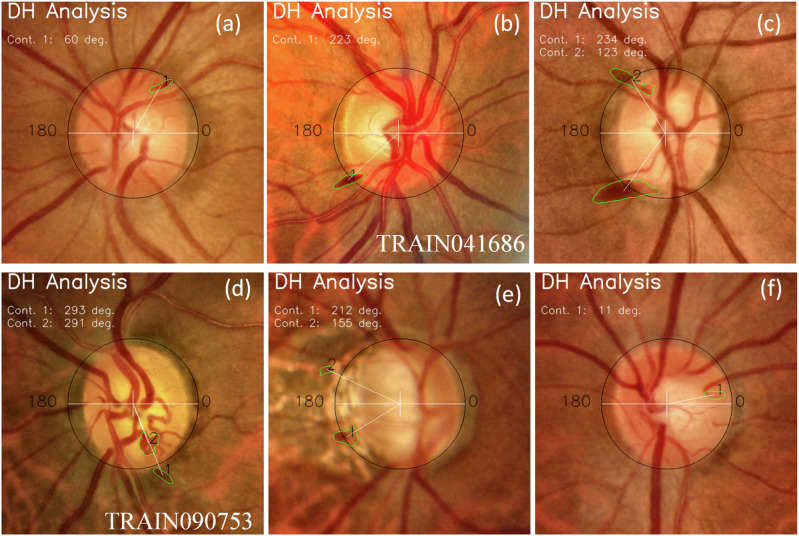
Examples of disc hemorrhage (DH) boundaries detected by the LWBNA-unet model. **a** DH at the superior-temporal (ST) region. **b** DH at the inferior-temporal (IT) region. **c** DH at both ST and IT regions. **d** DH at IT, with the marked area inside the disc not representing DH but a winding vessel. **e** Multiple markings, where the IT at the disc boundary likely represents real DH, but the ST marking is pigmentation. **f** A DH-negative case where a curved vessel resembles a hemorrhage.

Figure 7 shows examples of DH boundaries detected and marked on the fundus ROI images for both DH+ and DH- cases. Both the location and area of the hemorrhage can be estimated. For DH+ cases, the marked hemorrhage area is highly precise, even when it is much smaller than the overall image area (Fig. 7a). The model can recognize both single and multiple DH+ events in images captured from various cameras (Fig. 7b, c). In complex situations, such as in Fig. 7d–f, the model may detect multiple DH+ events that appear true at first glance. For example, in Fig. 7d, the DH marked in the inferior-temporal (IT) region just outside the disc is real, whereas the one inside the disc is false and represents winding or jumbled blood vessels. This image is from the AIROGS dataset (TRAIN090753) and is categorized as referral glaucoma (RG). The observed DH is located at the edge of the RNFLD, a common site of hemorrhage in glaucoma. False DH+ situations may arise when the algorithm analyzes DH− images, as shown in Fig. 7e, f. The model may output either no DH regions or multiple small spots (a few pixels) resembling DH+ in color. In most cases, these regions are easily eliminated. However, in certain situations, such as blood vessel winding (Fig. 7d, f) or local pigmentation (Fig. 7e), which resemble DH+ in color, size, location, and shape, distinguishing them can be challenging. This reflects the real-world challenges faced by ophthalmologists, where experience plays a crucial role. Thus, segmenting potential DH+ areas alone is insufficient for DH classification.

Therefore, the dataset used for training the DH classification model was reprocessed using the segmentation model to identify potential DH+ areas for retraining. The input to the model now consists of a six-channel image: three RGB channels for the original ROI image and three channels for the segmentation mask. The DH classification accuracy improved significantly, increasing from approximately 76% to 91%. The best-performing model (from ten training runs) achieved a specificity of ~94% and a sensitivity of 87% on the testing dataset, with an AUC of 0.9678 and a Youden index of 0.8108 at a threshold of 0.4004. Detailed results are summarized in Table 1. This improvement is likely due to the spatial information about DH provided to the classification model through the segmentation mask.

### Detection of retinal nerve fiber layer defects

**Fig. 8:**
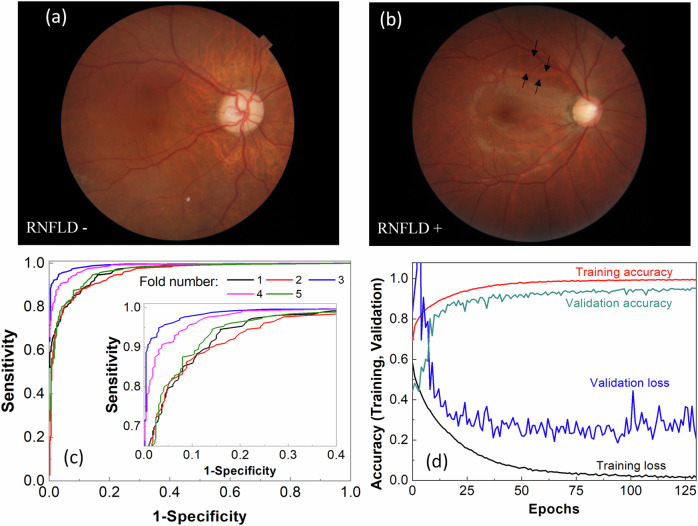
Detection of retinal nerve fiber layer defects (RNFLD) in macula-centered fundus images. **a** Example of an RNFLD-negative image. **b** Example of an RNFLD-positive image. **c** ROC curves for five models trained using a five-fold cross-validation scheme. The details of the curves are shown in the inset. **d** Training and validation curves for the best-performing model. The best-performing model achieved high prediction accuracy, with an AUC of 0.9933, a Youden index of 0.9182, and an accuracy of 95.97%.

RNFLD may be the earliest sign of glaucoma, often appearing long before visual defects become noticeable^13,14,42,43^. Detecting RNFLD in a fundus image can be challenging due to the insignificant intensity differences between the affected and normal regions. RNFLD typically appears as an arc extending from the optic disc toward the fovea. Few reports exist on the detection of RNFLD^44–46^, and there is currently no publicly available dataset. Creating such a dataset is challenging and prone to inconsistent labeling, leading to errors in model generalization and discrepancies in results across the training, validation, and testing datasets. Therefore, we created a dataset of 3721 fundus images and conducted a five-fold cross-validation training of the LWBNA-based model shown in Fig. 2a. Typical examples of input images (RNFLD− and RNFLD+) are shown in Fig. 8a, b. For demonstration purposes, the presence of RNFLD is marked by arrows in Fig. 8b, highlighting an arcade-like darker contrast starting from the edge of the optic disc.

Figure 8c shows the ROC curves for five models trained with a five-fold cross-validation scheme. The average prediction accuracy, AUC, and Youden index are 91.71 ± 3.21%, 0.9731 ± 0.159, and 0.7990 ± 0.0898, respectively. Although some variations exist between folds, they are not statistically significant. The best-performing model achieved a high prediction accuracy, AUC, and Youden index of 95.97, 0.9933, and 0.9182, respectively (Table 1). The training and validation curves for this model are displayed in Fig. 8d. The training loss consistently decreases, while the validation loss stabilizes and then begins to increase with increasing epochs. Differences in training and validation accuracy across epochs may be attributed to variations in labeling. Overall, the model’s training behavior on the created dataset appears consistent, and the detection results for RNFLD are promising.

### Glaucoma screening by multi-model AI-GS network

The AI-GS network combines the results of the aforementioned models using an FFCN to make a final screening decision. The inputs to the FFCN model consist of predictions (0/1) from the MTL_LWBNA-unet, disc assessments for cupping based on the FFCN model, DH predictions, and RNFLD predictions. The dataset for training the final FFCN model was created by combining all fundus images used for training and validation of the MTL, DH, and RNFLD detection models. A fundus image is labeled as positive (a case for referral to an ophthalmologist) if any prediction from the four models—direct DL, cupping-based FFCN, DH, or RNFLD—is positive. With this labeling scheme, the dataset contains 5062 fundus images labeled as negative (i.e., no glaucoma referral) and 4867 as positive. Compared to the direct prediction of glaucoma by the MTL_LWBNA-unet, the AI-GS network shows a slight improvement in five-fold cross-validation accuracy (96.28 ± 0.45% vs. 97.47 ± 0.04%). Table 1 summarizes the performance of the best-performing AI-GS network. Considering the 95% CI, the results for the AI-GS network and direct predictions by the MTL_LWBNA-unet do not differ significantly across the overall testing dataset when using the optimal threshold determined by the Youden index. However, in the real-world screening scenario using the “Miyagi Screening“ dataset, the AUC increased from 87.8% to 92.5%, and sensitivity improved from 56.5% to 80.5%, with only minor changes in specificity (~91%).

**Fig. 9:**
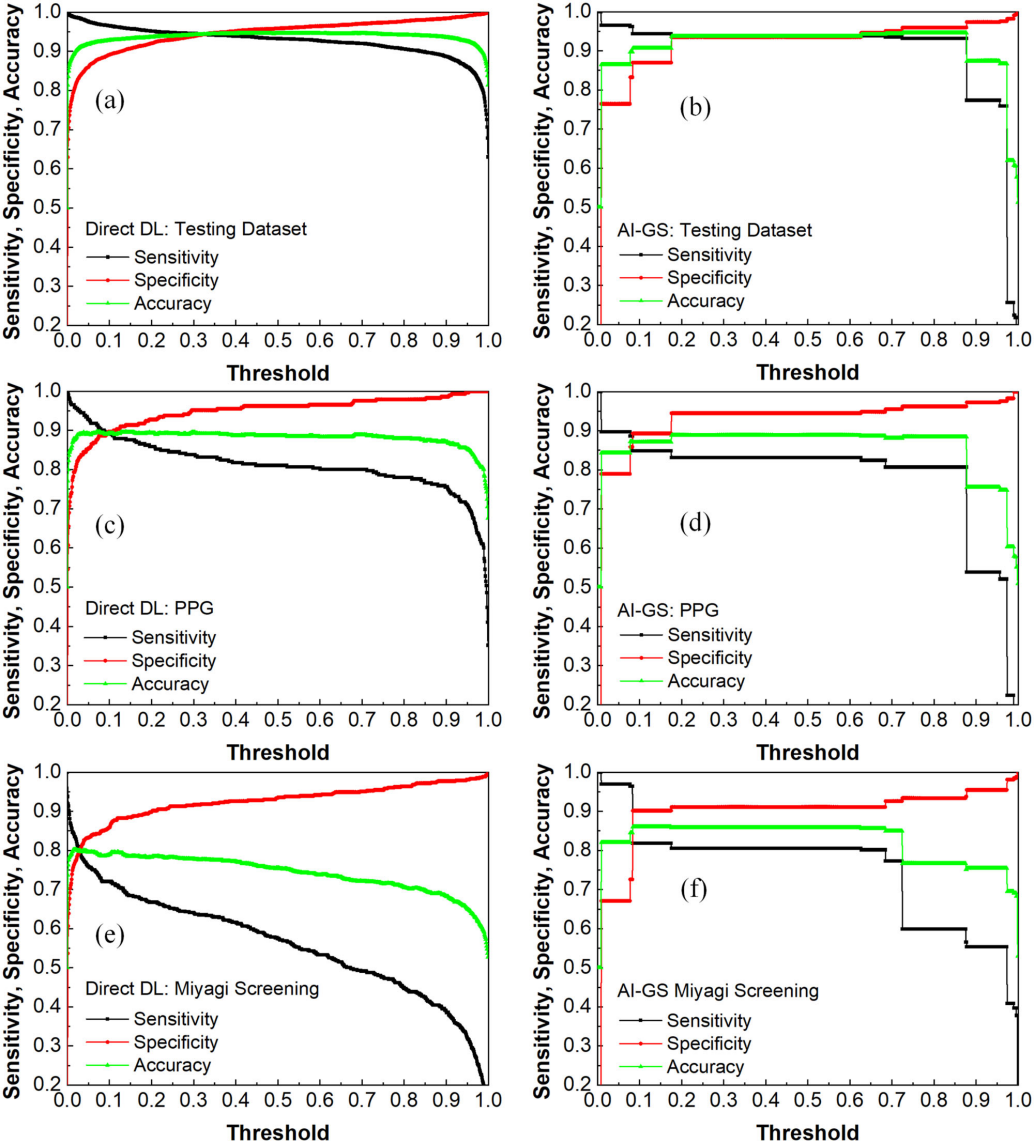
Dependence of sensitivity, specificity, and prediction accuracy on the binary classification threshold for a single model (MTL_LWBNA-unet) and the multiple-model AI-GS network. **a** Results from the MTL_LWBNA-unet model for the testing dataset. **b** Results from the AI-GS network for the testing dataset. **c** MTL_LWBNA-unet results for the PPG dataset. **d** AI-GS network results for the PPG dataset. **e** MTL_LWBNA-unet results for the Miyagi screening dataset. **f** AI-GS network results for the Miyagi screening dataset. The results demonstrate that the AI-GS network maintains consistent performance across a broader range of thresholds compared to the MTL_LWBNA-unet model.

To further understand the differences in direct glaucoma prediction between the single DL model (MTL_LWBNA-unet) and the AI-GS network, we performed Delong’s test^47^. This test assesses whether the differences in the AUCs between two ROC curves are statistically significant. The extremely low *p* values—1.7 ×10^−9^ on the Miyagi screening dataset and even smaller on the testing datasets (PPG, NTG, and POAG)—indicate highly significant differences. As presented in Table 1, the threshold values for each model were determined based on the Youden index, often considered the optimal cutoff for decision-making in diagnostic tests. To better understand the strengths and weaknesses of the current models, we analyzed and visualized sensitivity, specificity, and accuracy with respect to threshold levels. These analyses are shown in Fig. 9 for the entire testing dataset, Miyagi Screening, and separately for PPG, while corresponding curves for NTG, POAG, and the cupping-based dataset are included in Supplementary Fig. 3. It is noteworthy that these parameters vary with the threshold in the case of a single DL model-based binary classification (MTL_LWBNA-unet) but remain almost invariant over a wider range of thresholds for the AI-GS network. These results clearly suggest that the AI-GS network’s performance is robust and better suited for large-scale screening programs where consistent and reliable performance is crucial, irrespective of the disease stage or severity.

## Discussion

Based on the obtained results, it is evident that the AI-GS network can detect glaucoma from macula-centered fundus images, evaluate disc features, and identify early structural changes. However, a definitive diagnosis of glaucoma cannot be made without VF testing. Since PPG is a stage where structural changes are present but no VF abnormalities are observed, this model facilitates the detection of PPG. This is a significant achievement, as early detection of PPG is crucial in the era of 100-year lifespans, contributing to timely diagnosis and intervention in glaucoma management. Additionally, the efficient design, based on the LWBNA-unet model^34^ minimizes computational overhead to nearly one-third of the size of the widely used Unet^48^ model employed in image segmentation tasks. This represents a major step toward integrating glaucoma screening into telehealth, making faster and more accurate eye care accessible. While many models for glaucoma detection from fundus images have been reported^5,25,26,28,29,49–52^, they often provide limited quantitative data. The AI-GS network not only offers screening but also delivers detailed metrics on the optic cup, disc, and fovea, supporting ophthalmologists in diagnosis. This quantitative information can help in clarifying the relationship between structural changes and disease progression, which may differ across populations. This study emphasizes the importance of assessing the neuroretinal rim (NRR) and optic cup relative to disc size in glaucoma detection.

**Fig. 10:**
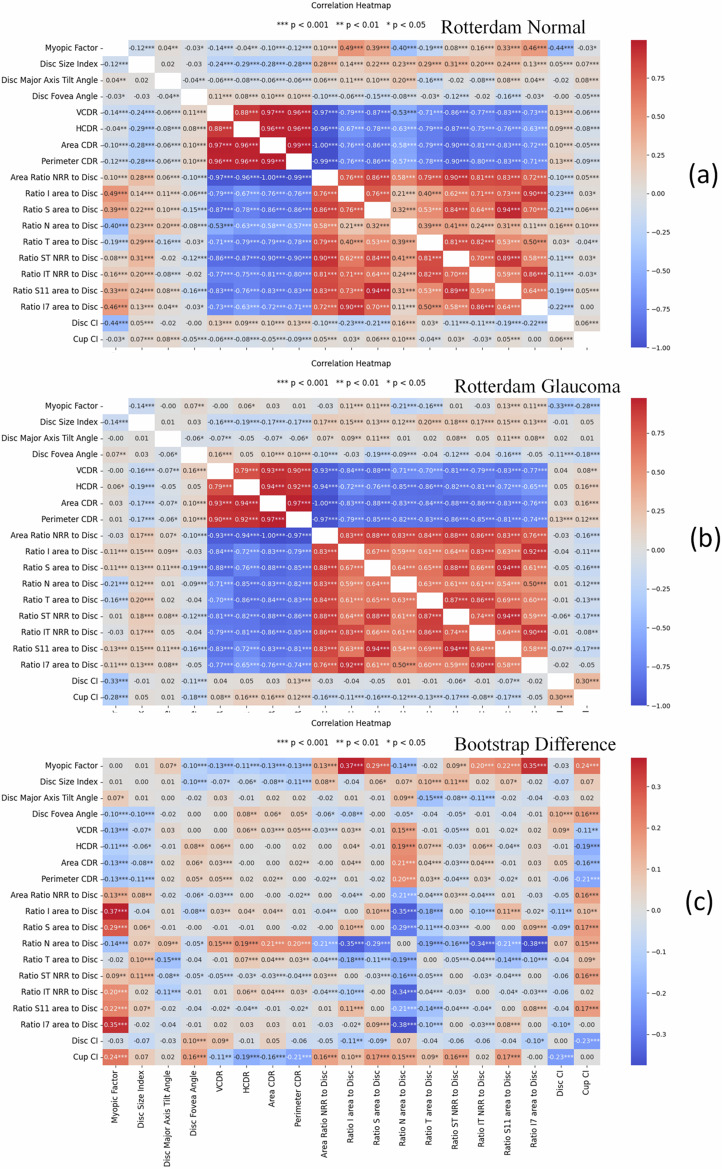
Correlation coefficient heatmap among different optic disc features in AIROGS dataset. **a** Normal. **b** Glaucoma. **c** Difference between normal and glaucoma, analyzed using the bootstrap method. These heatmaps, derived from the AI-GS network, provide insights into the relationships between various structural features in fundus images and their associations with disease and population characteristics. Star marks indicate statistical significance (i.e., *p* values).

In the current study, the AI-GS network aims to provide numerical data (disc and fovea assessments) and structural information (DH, RNFLD, and cupping), similar to that used in the labeling and diagnosis of glaucoma^36,53^. The MTL_LWBNA-unet model has demonstrated high accuracy in analyzing optic disc parameters from images captured by various cameras, with *D*_disc_ = 0.9601 ± 0.0202 and *D*_cup_ = 0.8895 ± 0.0609 (Fig. 4). Thus, the AI-GS network effectively facilitates the analysis of correlations among various optic disc features and their vulnerability to glaucoma. To demonstrate this, the training, validation, and testing datasets of AIROGS (NRG: 5540, and RG: 1436) and THU (Normal: 2726, NTG: 2511, and POAG: 1357) were used. Figure 10a displays the correlation heatmap between various features of the fundus images, including *p* values to denote their statistical significance. The optic disc in a normal eye is slightly oval, and it is reported that axial length is significantly associated with disc ovality, thus correlating with myopia^54^. Based on the optic disc shape, we defined the “Myopic Factor,” or the disc ovality index, as the ratio of the disc’s major axis to its minor axis, which increases with the severity of myopia. Our results indicate that myopia negatively correlates with the disc circularity index (Disc CI), as expected (Fig. 10a). As anticipated, myopia does not affect the disc-fovea angle^55^. The superior and inferior neuroretinal rim areas (Ratio S area to Disc, and Ratio I Area to Disc) exhibit a positive correlation with myopia, while the temporal and nasal areas are negatively correlated. The strength of the negative correlation is weaker in the temporal area compared to the nasal area. These correlation patterns significantly weaken in cases of glaucoma (Fig. 10b), potentially due to the preferential (non-uniform) loss of nerve fibers affecting the NRR area. Like the Myopic Factor, correlation among other retinal features can be understood from the heatmaps in Fig. 10. Bootstrap analysis of the difference in correlation coefficients between normal and glaucoma groups provides insights into how variable relationships differ across these groups. A difference close to zero implies that glaucoma does not significantly alter the relationship between certain parameters. Conversely, a non-zero difference could reveal insights into the mechanisms of glaucoma-induced changes. Figure 10c shows the heatmap of the difference in correlation coefficients among normal and glaucoma groups in the AIROGS dataset. Interestingly, the correlation difference between the Myopic factor and disc circularity index is not significant, indicating that glaucoma does not alter this relationship. However, the relationship between the cup circularity index and the Myopic factor has changed markedly, possibly due to irregular loss of retinal nerve fibers. Glaucoma appears to modify the relationship of nasal NRR with other disc features (Fig. 10c). A parallel analysis using the THU dataset (shown in Supplementary Figs. 4–8) reveals that the correlation heatmaps for normal and glaucoma groups (NTG and POAG) are similar to the AIROGS dataset, but there are noteworthy variations in the heatmap of correlation coefficient differences among NTG and POAG groups. The difference heatmap of THU-POAG is like that of AIROGS, suggesting prevalence of POAG glaucoma type. It is reported that among overall glaucoma cases worldwide, approximately 74% are of the POAG type^56^. This may explain the similarity between the glaucoma and POAG groups in these datasets.

**Fig. 11:**
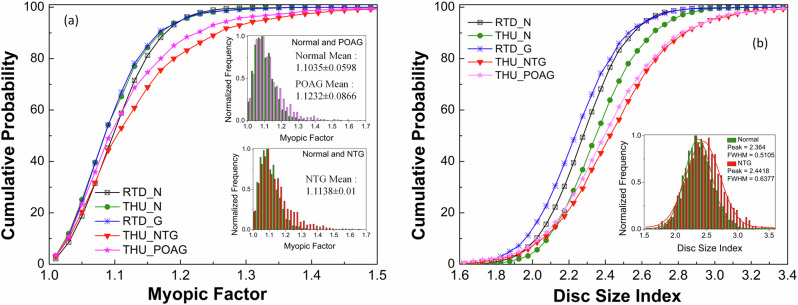
Cumulative probabilities obtained from histograms. **a** Myopic factor. **b** Disc size index for the AIROGS/Rotterdam dataset, including normal (RTD_N) and glaucoma (RTD_G) cases, as well as Tohoku University normal (THU_N), NTG (THU_NTG), and POAG (THU_POAG) cases. The results suggest that myopia and smaller optic disc size are risk factors for glaucoma in the Japanese population.

Further insights into the population-based differences and risk factors can be gained by analyzing the histograms of various retinal features. For example, the mean Myopic factor for the AIROGS dataset in normal (NRG = 1.11) and glaucoma (RG = 1.10) groups is similar and close to the reported mean (1.09)^57^. The THU dataset has the same mean value for normal (1.10), but it is slightly higher in POAG ( = 1.12) and NTG ( = 1.14) groups. Histograms, along with their cumulative probabilities (Fig. 11a) reveal that the prevalence of myopia is relatively high in the THU dataset (i.e. Japanese population), and it increases further in glaucoma. The results of Fig. 11a suggest that high myopia could be a risk factor for developing glaucoma in the Japanese population, especially the NTG type. These findings are consistent with the reported literature^58^. It was hypothesized that, parallel to the myopic stretching and thinning of sclera, the optic disc is also stretched, leading to an abnormally large and irregularly configured optic nerve head. This might be one of several reasons for the increased risk of glaucoma^59^. An increase in the negative correlation strength of the myopic factor with the disc circularity index for glaucoma in the THU dataset appears consistent. Another noticeable difference between these two datasets is in the disc size index (the ratio of disc-fovea distance to maximum disc diameter) among normal and glaucoma groups. Based on Fig. 11b, the prevalence of relatively larger disc size is high for glaucoma in the AIROGS dataset, whereas the opposite is true for the THU dataset. Both larger and smaller optic discs have been reported as potential risk factors for glaucoma. The higher prevalence of glaucoma in certain African populations, which have larger optic discs, led to the hypothesis that larger optic discs are prone to glaucoma^60^. It is reported that the pressure differential across the lamina cribrosa could produce increased deformation and displacement of central tissues in larger discs, leading to greater glaucoma susceptibility. In contrast, a larger disc has a larger lamina cribrosa area and more pores than a small disc, providing more space for nerve fibers and possibly reducing the likelihood of focal compression to axons, resulting in a lower risk of glaucoma compared to smaller optic discs. Eyes with smaller optic discs also have a smaller number of nerve fibers^61^. Although studies suggest that disc size alone might not be the risk factor^62^, the AI-GS network can provide further insights through rapid analysis of large-sized datasets from different population groups.

**Fig. 12:**
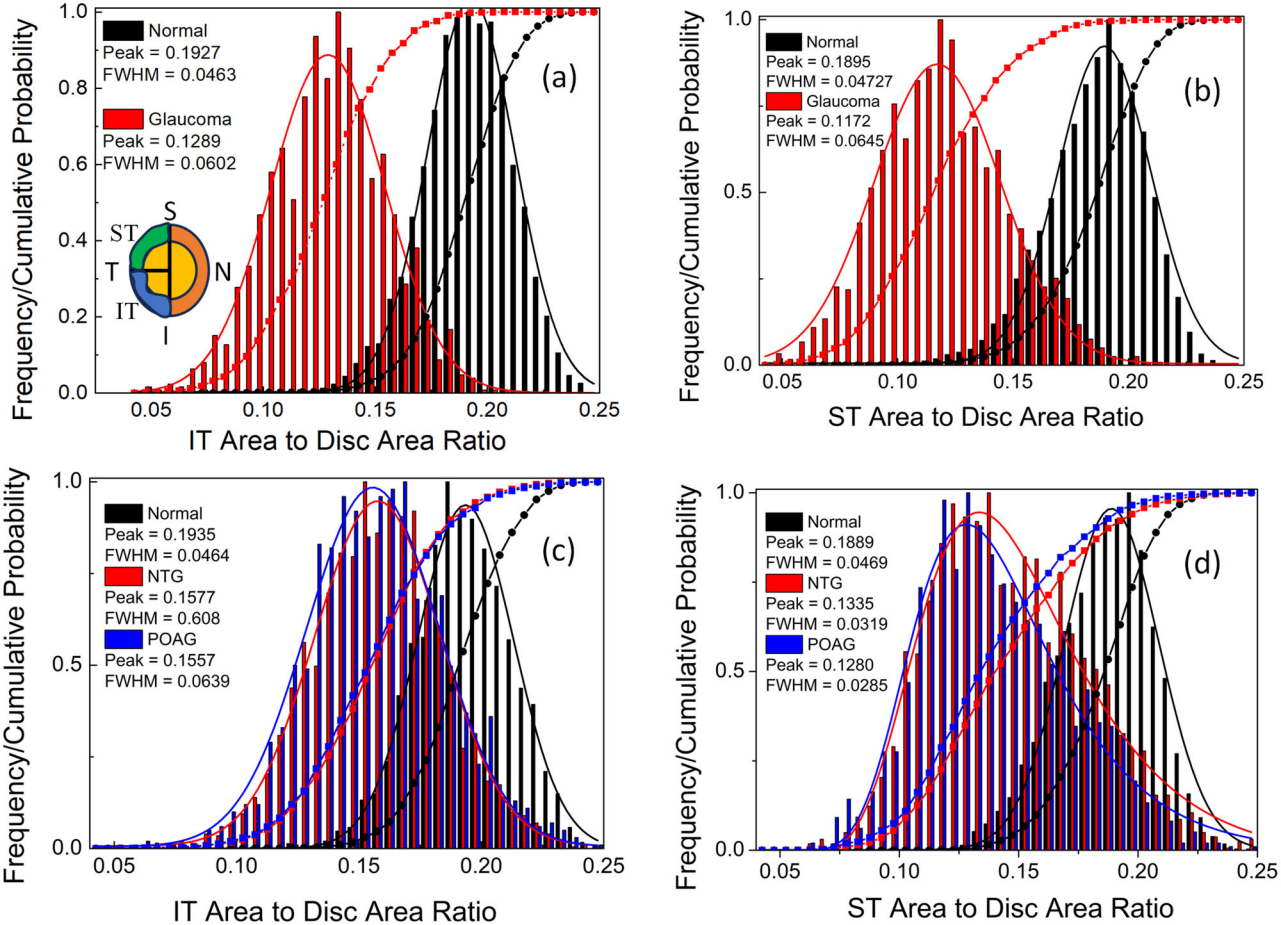
Normalized histograms and cumulative probabilities ratio. **a** Inferior-temporal neuroretinal rim (IT-NRR) area to disc area for AIROG. **b** Superior-temporal neuroretinal rim (ST-NRR) area to disc area for AIROG dataset. **c** IT-NRR to disc area for THU datasets. **d** ST-NRR to disc area for THU datasets. The results suggest that changes in the IT area of the optic disc are rapid and similar in both NTG and POAG cases, while changes in the ST region are slower and differ between NTG and POAG groups.

**Fig. 13:**
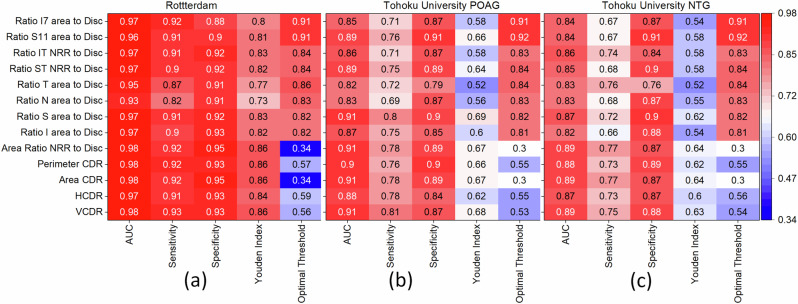
Glaucoma diagnostic ability of different optic disc parameters. **a** In the AIROGS dataset. **b** Tohoku University POAG dataset. **c** Tohoku University NTG dataset. As expected, the vertical cup-to-disc ratio (VCDR) demonstrates the highest diagnostic ability. The area under the curve (AUC) is higher for POAG compared to NT.

Figures 10 and 11 highlight differences in optic disc features and their correlations in normal, POAG, and NTG groups. These differences can be utilized for diagnostic purposes. Some of the clinically investigated parameters include vertical and horizontal CDR, the NRR area as a whole, and measurements in various disc sectors (such as inferior, superior, temporal, and nasal, or combinations thereof)^63^. The inferior (I) and superior (S) regions of the optic disc are often the first and most severely affected in glaucoma. These regions are used by ophthalmologists to differentiate glaucomatous damage from other types of optic neuropathies. Monitoring changes in the NRR area, especially in the inferior and superior temporal (IT and ST) regions, can be used to assess the risk of developing glaucoma. We analyzed the ratio of the IT- and ST-NRR area to the disc area for both the AIROGS and THU datasets. Histograms with cumulative probabilities are presented in Fig. 12. The distribution of the area for normal and glaucoma groups follows an almost normal distribution in both IT and ST regions for the AIROGS dataset. As expected, the peak area ratio of the distribution for normal and glaucoma groups is larger in the IT region compared to the ST. A narrower distribution, indicated by full width at half maximum (FWHM), in the IT region compared to the ST suggests that glaucoma produces rapid changes in the IT region. This finding is consistent with the reported literature^64,65^. The THU dataset, which is divided into POAG and NTG types, shows some interesting differences. The distribution in the IT region is close to normal for both NTG and POAG, but it deviates in the ST. This deviation is weaker for POAG and is similar to the AIROGS dataset. The changes in the area ratio are slower (i.e., indicating a wider distribution) for NTG compared to POAG (Fig. 12d). In POAG, elevated IOP is a significant risk factor, and the damage typically follows the characteristic pattern of glaucomatous optic neuropathy. Both the IT and ST regions of the NRR are affected, with the inferior region often showing damage first or more prominently. The pathophysiology of NTG is still not fully understood, but it is believed to involve factors beyond just IOP, including vascular and biomechanical aspects^66^. Vascular theories suggest that insufficient blood supply and vascular dysregulation play a role in NTG. These factors could contribute to the specific patterns of optic nerve damage observed in NTG, which may differ from those in other types of glaucoma. Nevertheless, significantly larger differences observed in the optic disc parameters for normal and glaucoma groups can be utilized for their separation. The ROC curves were plotted, and the AUCs for different parameters in the AIROGS and THU datasets are shown in Fig. 13. In general, all the optic disc parameters have high AUC (0.82–0.98). Vertical CDR, area CDR, and NRR normalized with the disc are the best for separating a normal eye from glaucoma. These results are consistent with the reported literature^63^.

The AI-GS network can diagnose glaucoma either through direct DL or by integrating early structural changes with DL predictions. Specifically, for diagnoses excluding PPG, the MTL_LWBNA-unet model offers superior sensitivity and specificity based on an optimal threshold detailed in Table 1. This threshold, however, varies across datasets such as PPG, Miyagi screening, POAG, and NTG, highlighting the necessity for dataset-specific adjustments (refer to Fig. 9 and Supplementary Fig. 3). The variation in fundus image quality and disease severity may influence the classification threshold, affecting the DL’s ability to detect disease-specific features. A potential solution involves developing a method to automatically adjust the threshold based on image quality and disease stages or creating a model whose predictions are robust across a broad threshold range. In this study, the AI-GS network achieved this by leveraging multiple models focusing on different aspects of glaucoma, maintaining stable prediction results across a threshold range of 0.1 to 0.65 for the analyzed datasets. This approach enhanced the network’s robustness against variations in image quality and disease stage.

In situations such as mass screenings or when resources are limited, ensuring a cost-effective testing protocol is important. Selecting a threshold that either maximizes sensitivity or balances sensitivity and specificity in a cost-effective manner is crucial. Prevent Blindness America^40^ recommends a sensitivity of at least 85% at a specificity of 95%. On the entire testing dataset, the AI-GS network showed ~94% sensitivity at 95% specificity and maintained similar specificity and sensitivity (~93–94%) across a wide range of thresholds. However, sensitivity drops to ~83–81% (specificity ~94–91%) for the PPG and Miyagi screening datasets. The AUC for detecting PPG using techniques like Pulsar, Flicker, OCT-disc and OCT-macular ranges from 0.663 to 0.842, whereas for early glaucoma, it ranges from ~ 0.851–0.907^67^. The AUC from the AI-GS network for PPG is ~ 0.9123, which is significantly high. Differences in labeling for PPG among the ophthalmologists may result in lower AUC/sensitivity because distinguishing between GS/PPG and normal is challenging, and no common consensus exists^68^. For instance, Deshpande et al.^13^ defined PPG as normal VF and one or more localized RNFLDs (on fundus image) associated with glaucomatous disc and IOP greater than 21 mmHg. Factors associated with glaucoma, such as DH, and a higher VCDR might be considered. Since glaucomatous morphological changes and VF disturbances are irreversible, examination should be performed regularly to identify disease progression as early as possible during the PPG stage (before the disease progresses to VF impairment)^11^. In such scenarios, where stringent detection is crucial and missing a case can have serious consequences, the priority is typically to maximize sensitivity.

The AI-GS network can be adjusted to meet different requirements as discussed above. For example, cases predicted as normal by AI-GS but with significantly high prediction probabilities for structural changes from sub-models (such as cupping, RNFLD, and DH) can be further classified as glaucoma suspects (GS). By changing the prediction from normal to GS when any of the following conditions are met—cupping probability >0.65, RNFLD probability >0.8, and DH probability >0.75—the sensitivity for detecting PPG can be increased further from 83.1% to 87.6%. The improvement is even more pronounced for the real-world screening dataset, where sensitivity increases from 80.5% to 93.0%. However, this comes at the cost of decreased specificity (Table 1). The glaucoma group of Miyagi screening includes subjects evaluated for 3, 2, or 1 year. The sensitivity of this adjusted AI-GS network for different groups was as follows: 0.9574 for Group 1 (consistent over 3 years), 0.8654 for Group 2 (2 years), and 0.75 for Group 3 (1 year). On the other hand, the sensitivity for these groups is 0.8571, 0.6442, and 0.625 for the AI-GS network, and 0.6165, 0.4327, and 0.2917 for the single DL binary classification model (MTL_LWBNA-unet), respectively. The cupping model, in particular, demonstrated the most consistent results, closely matching the assessments of the screening ophthalmologists. Sensitivity variations were 0.9449, 0.8173, and 0.8333 for Groups 1, 2, and 3, respectively, suggesting that screening ophthalmologists prefer to make decisions based on cupping.

To further assess the robustness of the AI-GS network compared to a binary classification model for real-world glaucoma screening, we reanalyzed 230 fundus images flagged as “Glaucoma” by the screening ophthalmologist but misclassified as “Normal” by the single DL binary classification model in the Miyagi screening dataset (Table 1). Three glaucoma experts (NT, TN, MS) independently reviewed the images without prior knowledge of AI predictions or screening outcomes (details in Supplementary Table 1). Results revealed significant variability in screening outcomes: the screening ophthalmologist (not necessarily a glaucoma specialist), prioritizing sensitivity to minimize missed positive cases, flagged all 230 images as glaucoma. In comparison, glaucoma experts flagged 206, 178, and 172 cases as glaucoma-positive, reflecting differences in criteria, clinical experience, and thresholds. The single binary classification model (MTL_LWBNA-unet) performed poorly, classifying all cases as “Normal,” highlighting its lack of sensitivity for real-world screening. In contrast, the AI-GS network correctly identified 127 glaucoma cases, and the Adjusted AI-GS model further improved to 193, closely matching glaucoma expert’s opinions. Supplementary Fig. 9 illustrates the AI-GS network’s superior sensitivity, accurately detecting critical features such as cupping, RNFLD, and DH, reclassifying images overlooked by the binary model, and identifying subtle pathologies missed even by the experts. Notably, the Adjusted AI-GS achieved higher agreement with all three experts, effectively complementing their evaluations and addressing diagnostic variability. Detection of these pathological features can help identify borderline cases often missed by screening ophthalmologists due to time constraints. Ophthalmologists may reconsider cases flagged by the AI-GS network. For instance, Supplementary Fig. 9d shows a case marked as normal by both the screening ophthalmologist and the binary classification model, but flagged as a referral by AI-GS, aligning with the glaucoma expert’s decision. These findings establish the AI-GS network as a sensitive and reliable tool for improving detection in challenging cases, addressing critical gaps in real-world glaucoma screening. We believe that integrating the AI-GS network into screening processes can enable general practitioners (GPs) to detect glaucoma at a level comparable to glaucoma experts, and in a significantly shorter time (less than a second).

The enhanced sensitivity for detecting initial structural changes may lead to the detection of DH, RNFLD, and cupping, which might not be related to glaucoma. Retinal diseases such as DR and branch retinal vein occlusion (BRVO) might exhibit hemorrhages and RNFLD. We have analyzed some of the fundus images from the NRG group in the AIROGS dataset, which were predicted to be GS. Supplementary Fig. 10 shows a fundus image where RNFLD in both the ST and IT regions is visible, but cupping and the DL-based glaucoma predictions are negative. In addition to RNFLD, the fundus image exhibits exudates and suggests a case of DR. In another example in Supplementary Fig. 11, DH in the ST-region without glaucomatous features was detected by AI-GS, classifying it as GS. However, some exudates and hemorrhages exist in an arcade position near the fovea, which might suggest a case of BRVO. Supplementary Fig. 12 does not exhibit any obvious features of retinal diseases, except for the visual appearance of choroidal patterns related to myopia. The AI-GS network detected DH in IT, which could be a risk factor for glaucoma (i.e., GS). The AI-GS network detected all features related to glaucoma (DH+, RNFLD+, glaucoma+, and cupping+) in Supplementary Fig. 13. It appears to be a case of glaucoma, but without a VF test, confirmation is difficult.

The present study may have some limitations in terms of the datasets and the results inferred from the analysis of glaucoma. About 71% of fundus images in the normal and 25% in the glaucoma category are from public datasets. Normal group images are non-glaucomatous but may include other retinal diseases or PPG cases (as noted, the AIROGS dataset^36,69^ was initially aimed for DR screening), which may affect retinal features and prediction accuracies. For the glaucoma group, detailed diagnostic procedures (such as perimetry, IOP) are unavailable. Although the diagnostic procedures of the THU-dataset are well supported by various measurements and ophthalmologists^70^ it is a hospital-based dataset where some bias in terms of disease severity may exist. The present study used macula-centered images to enhance the model’s ability to detect RNFLD and estimate the optic disc size index. Depending on the quality and resolution of the fundus images, there may be differences in optic disc parameters when estimated from disc-centered images. However, we believe macula-centered images are better suited for screening purposes, as they allow for the detection of other eye diseases beyond glaucoma. To fully assess the generalizability of the AI-GS network, testing it on additional screening datasets is necessary. Unfortunately, publicly available glaucoma datasets that include both macula-centered fundus images and comprehensive diagnostic information (such as IOP, VF, OCT, etc.) are currently limited. Despite these limitations, the AI-GS network demonstrated high accuracy in detecting glaucoma from the macula-centered images in the datasets used for this study. We believe that future data collection efforts from diverse population groups will help in addressing these challenges. Nevertheless, the AI-GS network’s architecture remains robust.

In conclusion, we have introduced an innovative AI-based Glaucoma Screening (AI-GS) network that leverages multiple DL models to accurately mimic the diagnostic approach of an ophthalmologist. Unlike traditional methods, the AI-GS network performs both segmentation and classification tasks, utilizing a hybrid approach that combines black-box predictions with conventional glaucomatous optic disc feature analysis. This dual methodology allows for the early detection of glaucoma indicators such as DH and RNFLD, crucial for preventing irreversible vision loss. On a testing dataset of over 8000 images, the AI-GS network achieves a detection accuracy of ~94%. A comparative analysis in real-world screening scenarios revealed a stark difference in sensitivity between direct DL-based approaches and the AI-GS network. This exceptional performance is attributed to the incorporation of multiple DL models trained on extensive datasets, featuring images captured by various cameras across different locations and ethnicities. The lightweight nature of AI-GS network (total memory size approximately 110 MB) enables its integration into everyday devices, such as smartphones and tablets. This capability is particularly transformative for remote or underserved communities, where access to healthcare resources and specialized ophthalmologic services is limited. By making accurate glaucoma screening accessible to individuals at their convenience, the AI-GS network stands to make a profound impact on global health, offering a scalable solution to prevent the progression of glaucoma and the consequent loss of vision. Future research could explore the integration of additional models trained on other eye diseases to broaden its screening capabilities.

## Methods

### Subjects and datasets

The present study adheres to the tenets of the Declaration of Helsinki, and the protocols were approved by the Clinical Research Ethics Committee of the Tohoku University, Graduate School of Medicine (No 2023-1-896). The ethics committee approved the study procedure, and all the methods were carried out in accordance with the relevant guidelines and regulations. Informed consent was obtained from all participants and/or their legal guardians.

Training of different DL models in the AI-GS network requires datasets and creating them is time-consuming. For example, the MTL_LWBNA-unet model requires labeling fundus images as glaucoma or normal, along with masks for the cup, disc, and fovea (Fig. 3a, b). It is hard to find such a large-sized dataset needed to train a robust DL model^9^. For the detection of RNFLD, fundus images of glaucoma patients are required. The detection of RNFLD is highly dependent not only on the pathological presence of the condition but also on several technical and patient-related factors. These include the type of fundus camera used, which affects image resolution and contrast, and age-related ocular conditions such as cataracts and corneal opacities. Furthermore, myopia-related changes, such as the tigroid/stripe pattern, can obscure RNFLD in fundus images. The situation is more complex for DH, as it is a time-dependent phenomenon (appearing/disappearing over time) and is not observed frequently. Separation of fundus images into normal/glaucoma or RNFLD-positive/-negative categories is relatively easier compared to the preparation of masks, which requires precise drawing of cup and disc boundaries. Additionally, mixing images obtained from different cameras and facilities can be beneficial for making a DL model more resilient to changes in domain or modality. In the present work, we searched various publicly available datasets^36,37,39^, as explained in Table 3, and mixed them with the fundus images of glaucoma/normal patients diagnosed at Tohoku University Hospital^70^. For the present study, we considered fundus images centered on the fovea/macula. For the subdivision of datasets into PPG, POAG, and NTG categories, non-glaucoma cases were randomly selected to create balanced datasets. For instance, the testing dataset includes 1373 fundus images of the POAG type. An equal number of non-glaucoma images were also randomly chosen from a pool of 4185 images, as detailed in Table 3.

**Table 3:** Details of fundus image datasets used for training, validation, and testing of DL models

	Dataset used for training of multi-task deep learning model		
Source	Non-glaucoma	Glaucoma	Comments
REFUGE	1079	118	Selected from 1200 images in training, validation and testing datasets
EyePACS AIROGS	2284	1544	Selected from ~ 3270 images of glaucoma and 98173 non-glaucoma referrals. For non-glaucoma images with file size ≥ 1500 kb were considered
ODIR	0	99	
Tohoku University	1832	1192	Labeling by glaucoma experts using fundus, OCT, IOP and visual field
Total	5195	2953	
	Dataset for testing of multi-task deep learning model		
EyePACS AIROGS	3284	0	
Tohoku University	901	4185	PPG = 290; POAG = 1373; NTG = 2522
Total	4185	4185	
	Real-world screening dataset		
Miyagi	10767	529	Labeling of fundus images by ophthalmologist
	DH Binary classification		
	DH−	DH+	ROI: Images cropped around the disc center with ~2*diameter of Disc
Training	1127	918	Public dataset: 32 DH− & 43 DH+ from Rotterdam
Validation	252	205	Public dataset: 99 DH+ from Rotterdam
Testing	179	180	
	RNFLD Binary classification		
	RNFLD−	RNFLD+	Fivefold-cross validation; Images from public dataset: 1016 RNFLD− & 191 RNFL+
Training/validation	2438	1283	
	DH segmentation (DH+ and mask)		
	Original	After augmentation	
Training	1408	6837	ROI: Images cropped around the disc center with ~2*diameter of Disc
Validation	296	1470	

*REFUGE* Retinal Fundus Glaucoma Challenge, *AIROGS* Artificial Intelligence for Robust Glaucoma Screening, *ODIR* Ocular Disease Intelligent Recognition, *DH* disc hemorrhage, *RNFLD* retinal nerve fiber layer defects, *ROI* region of interest, *RNFLD* retinal nerve fiber layer defect.

#### EyePACS-AIROGS (Rotterdam) dataset

The Rotterdam EyePACS Artificial Intelligence for Robust Glaucoma Screening (AIROGS) dataset^36^ is a comprehensive collection of color fundus images from various subjects and sites, designed for glaucoma detection and classification. It includes 113,893 images obtained from 60,357 subjects across approximately 500 different sites, featuring individuals of diverse ethnic backgrounds. Images are labeled as either “referable glaucoma (RG)” or “non-referable glaucoma (NRG)” based on adherence to the guidelines established by the International Council of Ophthalmology. From this dataset, we used 1544 images of RG and 2284 of NRG in our training dataset. In the testing dataset, 3284 images of NRG are also included. The selection was based on the fundus image file size (>1.2 MB) and the presence of both optic disc and fovea.

#### Retinal Fundus Glaucoma Challenge (REFUGE) dataset

The REFUGE dataset^37^ was introduced as a satellite event at the 2018 MICCAI conference. This dataset comprises 1200 fundus images, each accompanied by masks specifying the positions (x, y pixel coordinates) of the optic cup, disc, and fovea. The images were acquired using two distinct camera models: Canon CR2 (400 images for testing and 400 for validation) and Zeiss Visucam 500 (400 images for training). Among these 1200 images, 120 are labeled as glaucoma. We used this dataset for segmenting the cup, disc, and fovea boundaries as well as for training the MTL_LWBNA-unet for glaucoma classification.

#### Creation of cup, disc, and fovea masks

To create a dataset for training the MTL_LWBNA-unet model, we initially trained a segmentation model, the “LWBNA-unet”^34^, using the publicly available REFUGE dataset (Table 3), which provides masks and positional information for the optic cup, optic disc, and fovea. To enhance the model’s robustness to domain shifts, we combined the original training and testing datasets into a new training set, while retaining the original validation dataset for evaluating the LWBNA-unet model. For detecting the fovea center, we experimented with both binary circle masks and regression-based outputs. While the model produced good segmentation results for the optic cup and disc, it frequently failed to accurately detect the fovea center. This issue likely arises because multiple regions in the fundus image exhibit pixel intensities similar to those of the fovea, making precise localization challenging.

To address this issue, we introduced spatial context to simulate the variations in pixel intensity around the fovea^71^. A 2D Gaussian distribution was used to generate a heat map of the fovea within the binary mask of the optic cup and disc. The 2D Gaussian kernel is centered on the fovea, with pixel intensity decreasing as the distance from the center coordinates increases. The spread and concentration of the heat map can be controlled by adjusting the standard deviation (*σ*). The equation below is used to generate the heat map of the fovea^71^.

1 $$H(x,y)=\exp \left(-\frac{{\left(x-\alpha \right)}^{2}+{\left(y-\beta \right)}^{2}}{{2\sigma }^{2}}\right)$$

Where (*α*, *β*) is the center of the fovea, and (*x*, *y*) denotes the coordinates of an image vector whose dimensions are 20% of the square-shaped fundus image.

The fovea masks generated using Eq. (1), along with the disc and cup masks, were used to train the LWBNA-unet model six times. We then applied the LWBNA-unet model, trained on the REFUGE dataset, to generate disc, cup, and fovea masks for fundus images captured by a Topcon fundus camera equipped with DRI OCT. This allowed us to create an extended dataset by combining REFUGE dataset images with images from the Topcon camera. While the number of validation images remained the same (400), the training dataset increased to 2058 images. Data augmentation techniques, such as brightness adjustments, minor cropping, and rotation, further expanded the training set to 6174 images, while the validation set grew to 1173 images. The LWBNA-unet model was trained six more times.

#### Chaksu dataset

It is a publicly available dataset^39^, encompassing a total of 1345 fundus images captured by three different commercial cameras (Remidio fundus on Phone, Forus 3Nethra Classic, and Bosch handheld cameras). The dataset has been divided into distinct training and testing subsets. In the context of this study, particular attention was directed towards the images from the training subset, specifically those acquired using the Remidio Fundus on Phone and Bosch handheld cameras. Images from the Forus camera were excluded due to cropping issues. We used this dataset to test the segmentation accuracy of the optic cup and disc. Notably, while the MTL_LWBNA-unet model was trained on fundus images centered around the fovea, the images in the Chaksu dataset were centered around the optic disc. The typical method of estimating CDR involves detecting both the optic disc and cup; however, the AI-GS network encounters errors if it fails to detect the fovea’s position, a crucial element for estimating the optic disc size index. As a result, we selected 500 images from the initial 955, basing our choice on visual image quality and the algorithm’s ability to detect the cup, disc, and fovea positions without error. It is worth mentioning that if required, the algorithm can be tailored to obtain parameters related to the optic disc only (without fovea), facilitating the processing of disc-centered fundus images.

#### Tohoku University Hospital (THU) dataset

Data from patients who visited Tohoku University Hospital in the last 10 years were considered for the present study. Glaucoma/Normal diagnoses relied on multiple tests (OCT, fundus photography, VF, IOP) performed by the specialists and their clinical characteristics have been reported previously^70,72,73^. For PPG cases, the optic nerve showed glaucomatous damage, but VF defects were not yet detectable by standard perimetric tests. To eliminate medication effects, we used fundus images from patients’ initial visits. We included 1832 fundus images of normal eyes and 1192 images of glaucoma in the training dataset. The testing dataset includes 901 images of normal and 4185 images of glaucoma. Out of 4185 glaucoma images, 2522, 1373, and 290 were of NTG, POAG, and PPG types, respectively.

#### ODIR (Ocular Disease Intelligent Recognition) dataset

The ODIR dataset^38^ is a structured ophthalmic database designed to facilitate research in ophthalmology and fundus image analysis. This dataset encompasses data from 5000 patients, including age, color fundus photographs from both left and right eyes, and diagnostic keywords provided by medical professionals. The dataset can be employed for multi-label, multi-disease classification of fundus images, encompassing conditions like diabetic retinopathy, glaucoma, cataract, age-related macular degeneration, hypertension, pathological myopia, and other abnormalities. In the current study, we only included the fundus images labeled as glaucoma in the training dataset. This selection was made to avoid a significant class imbalance (Glaucoma/non-glaucoma).

#### Real-world screening data from Miyagi Glaucoma Screening

For testing the performance of the AI-GS network on real-world glaucoma screening data, we used images from the Miyagi Glaucoma Screening checkup. We received images captured by a Canon fundus camera over three consecutive years (2020–2022). All participants in this dataset are Japanese. There were ~11,000 right eyes marked as normal for three consecutive years of checkup. We analyzed 10,767 fundus images of normal right eyes from the testing year of 2020. The demographic breakdown in the normal group shows a male-to-female ratio of 1.25. The mean and std of age are 54.08 ± 15.06 years, and the BMI is 23.74 ± 3.79. Other health metrics include systolic blood pressure at 129.77 ± 19.81 mmHg, diastolic blood pressure at 78.48 ± 12.40 mmHg, HDL levels at 63.90 ± 16.50 mg/dL, LDL levels at 122.68 ± 31.33 mg/dL, and HbA1c at 5.72 ± 0.57. For glaucoma detection, we considered 529 fundus images from 209 subjects over 3 years. Among these, 133 subjects were present during all 3 years, 52 for 2 years, and 24 for only 1 year. The male-to-female ratio in this group is 1.09, with an average age of 61.87 ± 10.36 years, BMI of 24.15 ± 4.22, and similar ranges for other health parameters [systolic/diastolic blood pressure (mmHg) = 132.77 ± 20.07/79.87 ± 12.16, HDL/LDL = 64.40 ± 15.97/122.88 ± 29.16, and HbA1c = 5.89 ± 0.52]. Labeling for glaucoma and normal fundus images was performed by screening ophthalmologists.

#### Dataset for DH detection and segmentation

Publicly available datasets for DH remain scarce. Considering this, we constructed a training dataset comprising fundus images with both positive (DH+ = 918) and negative DH instances (DH− = 1127). For validation, the dataset contains 252 images of DH− and 205 for DH+. Both the testing and validation datasets have multiple images of the same eye (both eyes), but none of the images of a single patient are present in both the datasets. Given that the region of interest (ROI) pertains to the area around the optic disc, the original images necessitate cropping. To achieve this, the contour of the optic disc, detected by MTL_LWBNA-unet, was fitted to a circle, allowing determination of its radius and center (i.e., disc center). Subsequently, the fundus images were cropped into square shapes, with dimensions approximately two times the disc diameter. These ROI-selected images were resized to 512 × 512 pixels and subjected to the CLAHE algorithm to adjust image intensity and contrast. In the training of the DL model for detecting DH, both the original ROI-selected images and the images processed with CLAHE were employed to account for variations in image quality. This augmentation strategy effectively doubles the volume of images utilized for training (4090) and validating (914) the model. We also created a dataset for model testing, consisting of fundus images categorized into two groups: DH− (179 images) and DH+ (180 images). Importantly, this dataset contains only single images of individual eyes.

For the segmentation of DH+ areas in fundus images, we selected 1704 images with DH+. As mentioned above, these were cropped to select images with the ROI. Binary masks for DH were created manually. The images were split into training (1408 images) and validation datasets (296 images). The dataset contains multiple images of the same eye recorded at different time intervals, but the same eye image is not present in both the training and validation datasets. By using image data augmentation (random rotation, cropping, stretching, changing brightness/contrast, etc.) the number of images was increased to 8307 (training: 6837, and validation: 1470).

#### Dataset for RNFLD detection

There is currently no publicly available dataset for RNFLD detection. Creating such a dataset is challenging, and it is likely to have inconsistent labeling due to unclear presence in fundus images. We created a dataset of 3721 fundus images, comprising 2438 RNFLD-negative and 1283 RNFLD-positive cases. The dataset was divided into five sets for five-fold cross-validation training, ensuring a consistent ratio of RNFLD-positive and RNFLD-negative images (20%). To enhance the robustness of the trained model, we employed data augmentation and increased the number of images to 14,884.

### Implementation of DL model

A workstation (DELL Precision 7820 Tower) equipped with an NVIDIA RTX A6000, 48 GB GPU, and 192 GB RAM was used for training and validation of DL models shown in Fig. 2. The open-source programming language, Python 3 backed by Keras API integrated in Tensorflow 2, was used. Details of the parameters used for training the LWBNA-unet model for segmentation have been reported in our previous paper^34^. For the training of classification models, we used categorical cross-entropy as the loss function.

### Evaluation metrics

The Dice coefficient (*D*) is utilized specifically to evaluate the performance of an image segmentation model^34^. It quantifies the similarity between the predicted segmentation and the ground truth, with values ranging from 0 (indicating no overlap) to 1 (indicating perfect overlap). For evaluating the performance of classification models, several different metrics are employed. Prediction accuracy is the ratio of correct predictions (glaucoma and normal) to the total number of cases, providing a general measure of the model’s performance. Sensitivity, or the true positive rate, is the ratio of correct glaucoma predictions to the total number of glaucoma cases, assessing the model’s ability to correctly identify positive instances. Specificity, or the true negative rate, is the ratio of correct normal predictions to the total number of normal cases, evaluating the model’s ability to correctly identify negative instances. The Receiver Operating Characteristic (ROC) curve is important for understanding the performance of a binary classification (glaucoma/non-glaucoma). It visualizes the trade-off between the true positive rate (sensitivity) and the false positive rate (1 − specificity) across different classification thresholds. The area under the ROC curve (AUC) offers an aggregate measure of performance across all possible classification thresholds, reflecting the model’s ability to distinguish between classes. An AUC of 0.5 indicates random guessing and 1.0 is for perfect classification. The optimum threshold value for classification decisions is often determined using the Youden Index, which maximizes the difference between the true positive rate and the false positive rate, effectively balancing sensitivity and specificity. A higher value of the index indicates better model performance, with 1 being the best possible value (achievable when TPR is 1 and FPR is 0).

To systematically assess differences in correlation patterns between glaucoma and normal groups within our datasets and determine confidence intervals (CIs), we developed a robust, Python-based analytical framework using “SciPy” for statistical analysis^74^. Our primary objective was to understand variability in different metrics (AUC, sensitivity, specificity, and Youden Index) and identify potential biomarkers or risk factors associated with glaucoma. For computing CIs around these metrics, we employed a bootstrap resampling technique using Python. This technique involves generating multiple bootstrap samples from the original data, calculating the desired metrics for each sample, and then determining the empirical distribution of these metrics. The CIs are subsequently extracted from these distributions by selecting the appropriate percentile values based on the desired confidence level (typically 95%), ensuring a robust estimation that accounts for the inherent variability in the data. Separate correlation matrices were generated for each group, employing the Seaborn library for heatmap visualizations. To ensure the appropriateness of our correlation analysis, we conducted preliminary tests of normality on our datasets using SciPy^74^. Based on these assessments, Pearson correlation was chosen for datasets that conformed to normal distribution, while Spearman rank correlation was utilized for datasets that did not, accommodating both linear and monotonic relationships. A critical aspect of our methodology is leveraging bootstrap techniques to estimate the variability and statistical significance of the observed correlation differences. By generating thousands of bootstrap samples, we constructed empirical distributions of correlation differences, deriving statistical estimates and CIs. This non-parametric approach is advantageous as it does not assume a specific underlying distribution, enhancing the robustness of our findings. Bootstrap-derived estimates were integrated into heatmap visualizations, offering a statistically grounded comparison of correlation patterns characteristic between different datasets. *p* values play a crucial role in our statistical analysis, serving as a tool to quantify the evidence against a null hypothesis. Conventional significance levels (***p < 0.001, **p < 0.01, *p < 0.05) were adopted to annotate heatmaps. ***p < 0.001 to indicate an extremely strong likelihood that the observed correlation difference is not due to random chance.

## Supplementary information

Supplementary Information

## Data Availability

The present study used fundus images from publicly available datasets (AIROGS, REFUGE, ODIR, and Chaksu) as well as from Tohoku University Hospital. Public datasets can be accessed through their respective websites, while images from Tohoku University cannot be made publicly available due to patient privacy concerns.

## Code availability

The code for training segmentation, classification, and multi-task learning models will be available at the following GitHub repository: https://github.com/parmanandsharma/Lightweight_AI.
