# Supplementary material for: A hybrid multi model artificial intelligence approach for glaucoma screening using fundus images
Source: NPJ Digit Med. 2025 Feb 27;8:130. doi: 10.1038/s41746-025-01473-w (PMC11868628; doi:10.1038/s41746-025-01473-w)
Supplement: Supplementary file 1 — Supplementary Information [file 41746_2025_1473_MOESM1_ESM.pdf]

Supplementary information

A Hybrid Multi Model Artificial Intelligence Approach for Glaucoma Screening Using Fundus Images

Parmanand Sharma, Naoki Takahashi, Takahiro Ninomiya, Masataka Sato, Takehiro Miya,
Satoru Tsuda and Toru Nakazawa

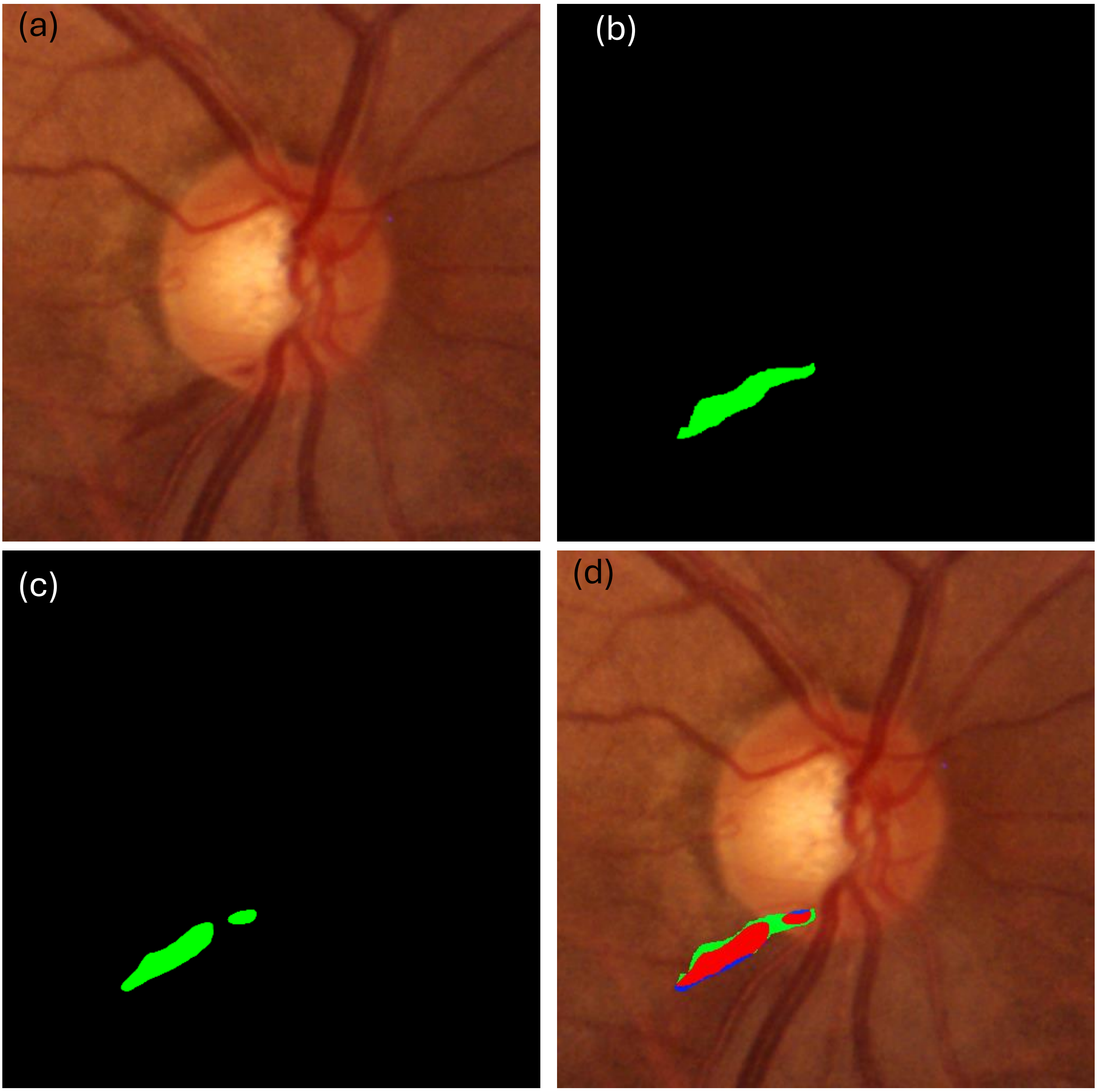

Supplementary Figure 1 | Example demonstrating the precision of LWBNA-unet for segmentation of DH in fundus image. a Image cropped around the optic disc, **b.** DH mask i.e., ground truth used for training of the model. **c** DH mask predicted by the model. **d** Overlap of manually made (green) and by the model (blue) on the fundus image. Red color represents the overlapping area of both the masks. A careful observation of cropped image suggest DH color is fainted/disappear at the optic disc boundary, and it is more precisely marked by the DL model.

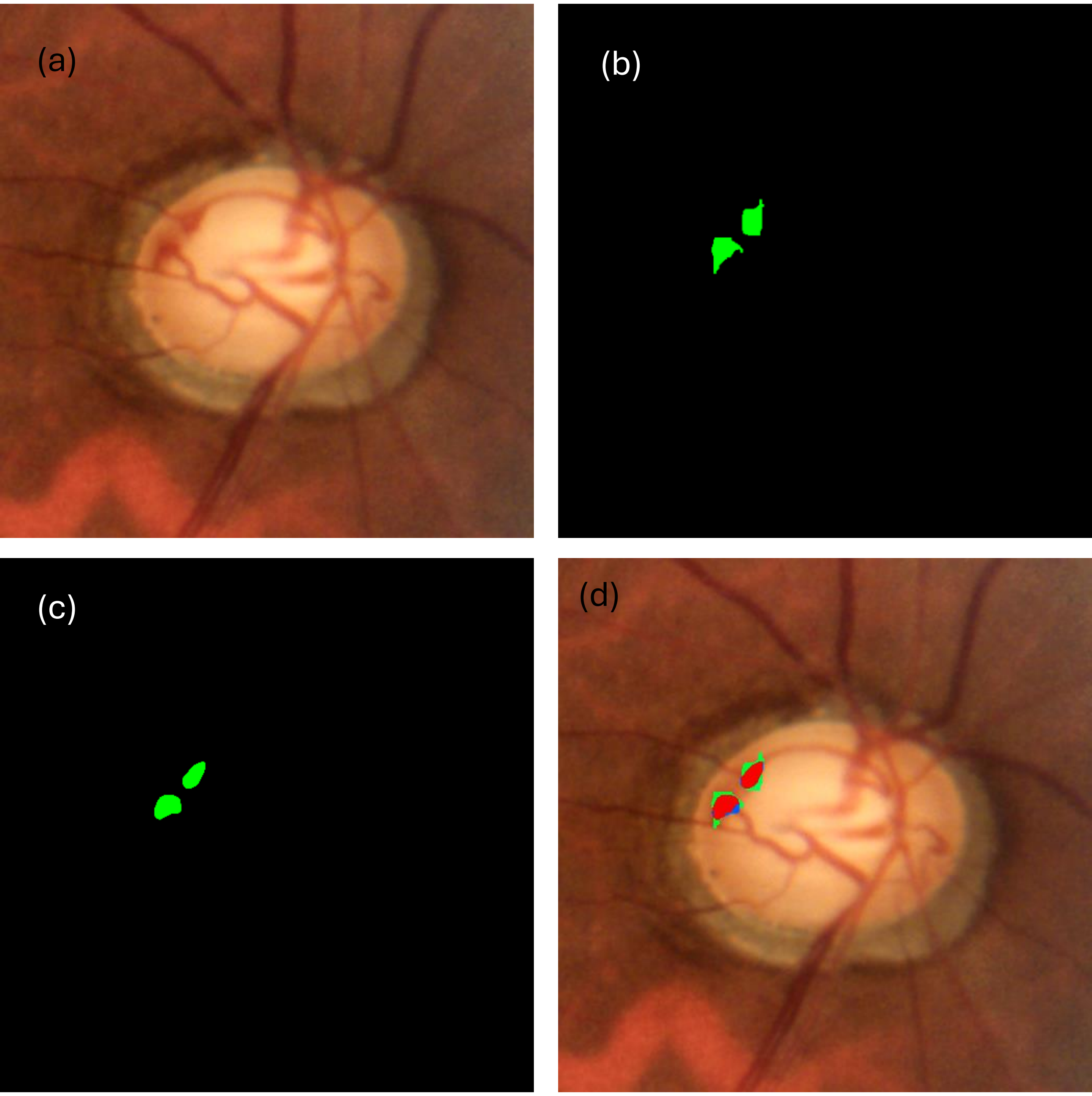

Supplementary Figure 2 | Another example demonstrating the precision of LWBNA-unet for segmentation of DH in fundus image. **a** Image cropped around the optic disc. **b** DH mask i.e. ground truth used for training of the model **c** DH mask predicted by the model. **d** Overlap of manually made (green) and by the model (blue) on the fundus image. Red color represents the overlapping area of both the masks. A careful observation of cropped image suggests manually drawing of mask is oversized.

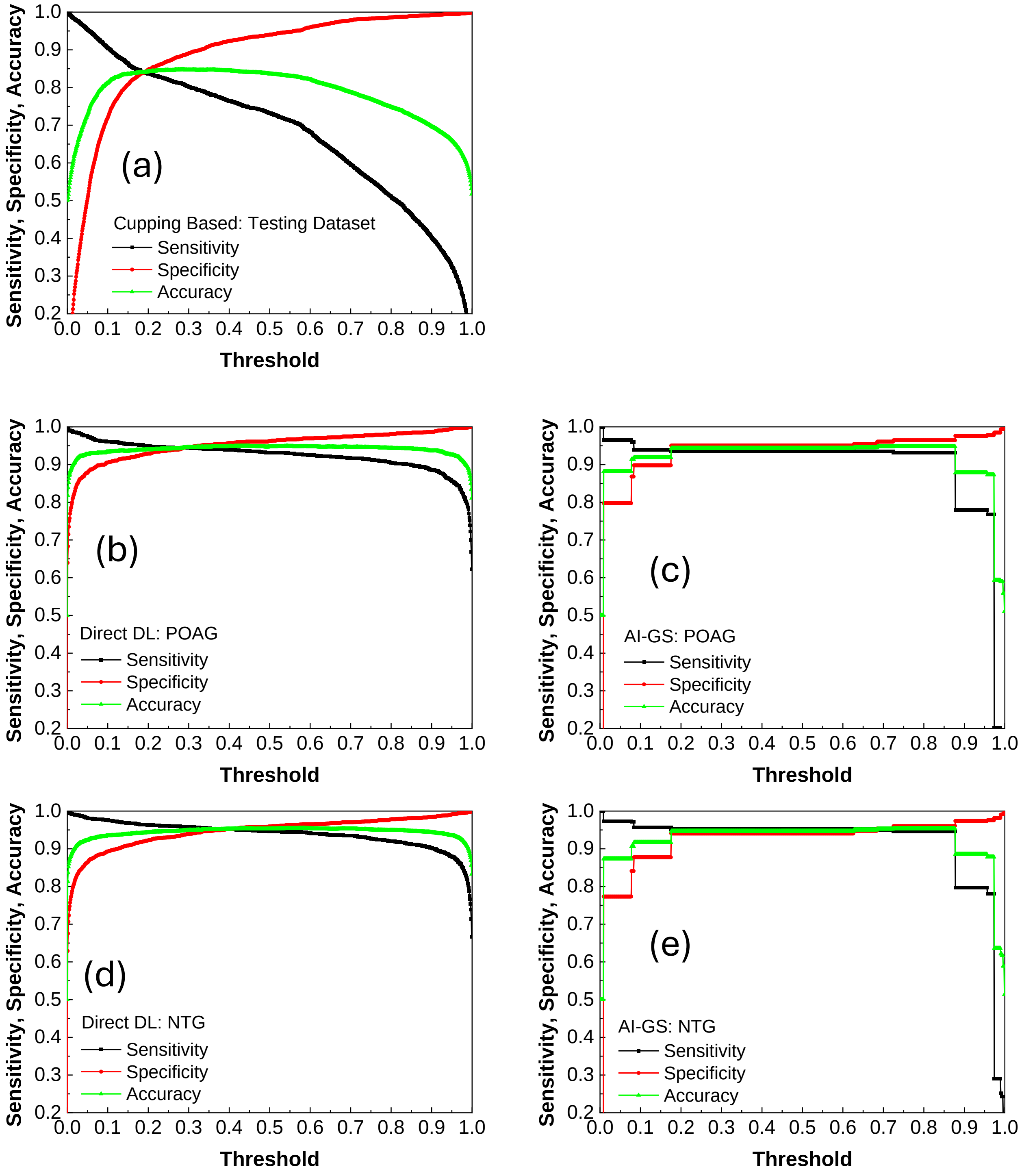

Supplementary Figure 3 | Variations in sensitivity, specificity, and prediction accuracy across different thresholds for MTL_LWBNA-unet and AI-GS Network. **a** Cupping based FFCN model utilizing numerical parameters determined from the segmented images of optic cup, disc and fovea by the MTL_LWBNA-unet for the testing dataset. **b**, and **d** Show the results for MTL_LWBNA-unet applied to the POAG and NTG datasets, respectively. **c**, and **e** depict the results for the AI-GS network across the same datasets: POAG and NTG.

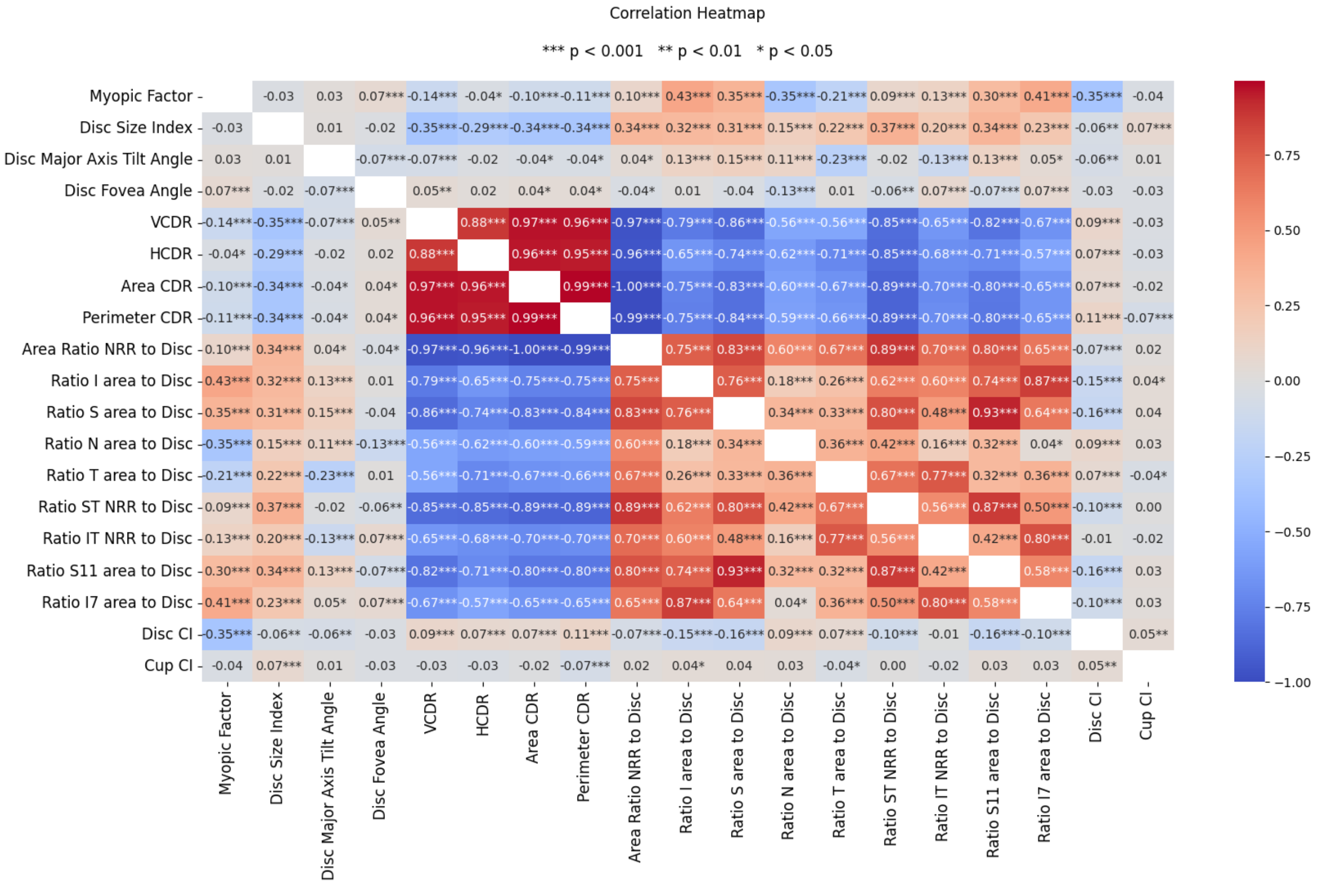

Supplementary Figure 4 | Correlation coefficient heatmap among different optic disc features in Tohoku University dataset of normal eyes. The correlation heatmap is similar to the AIROGS dataset, as discussed in the manuscript.

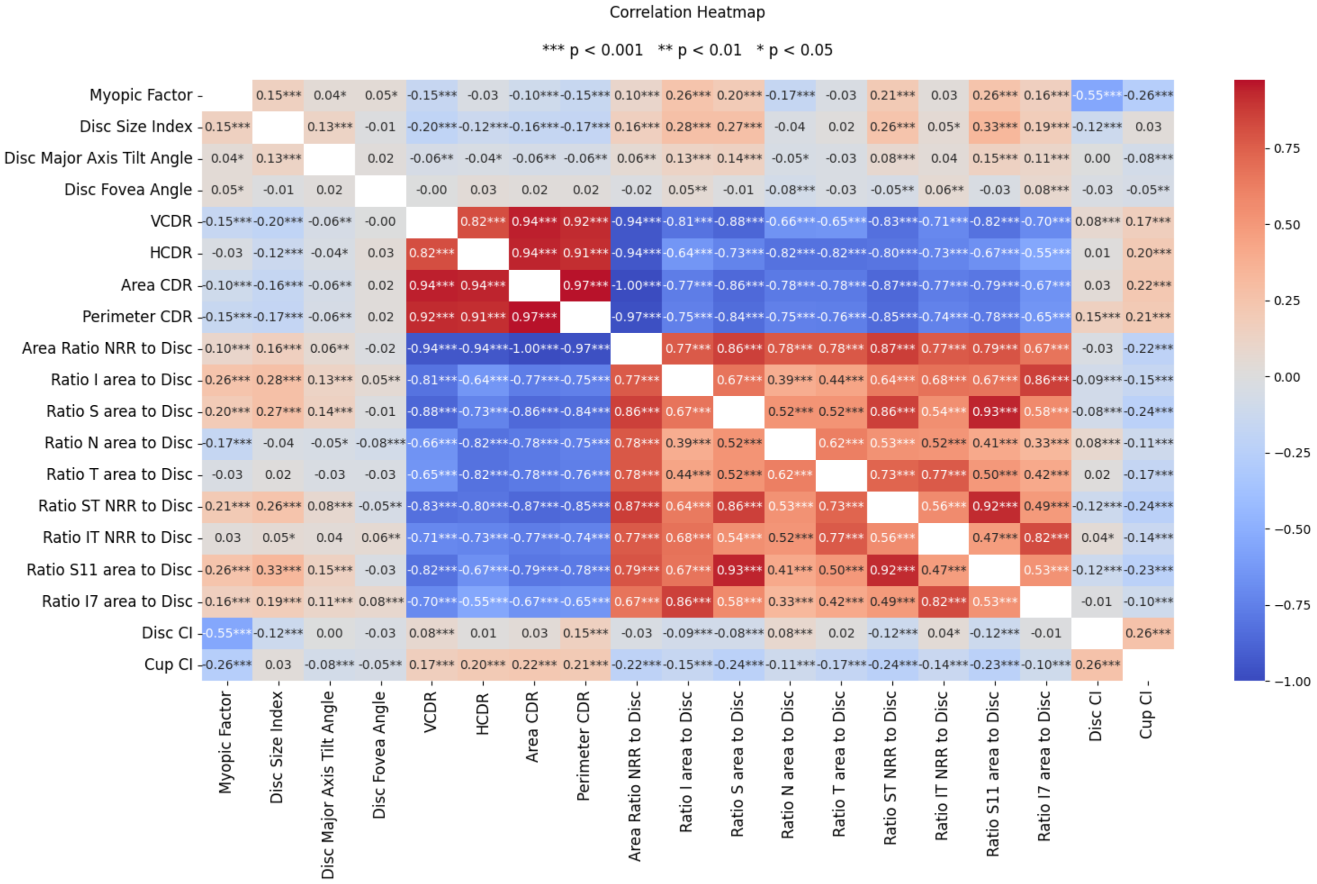

Supplementary Figure 5 | Correlation coefficient heatmap among different optic disc features in Tohoku University dataset of normal tension glaucoma (NTG) eyes. The correlation heatmap is similar to the AIROGS dataset, as discussed in the manuscript.

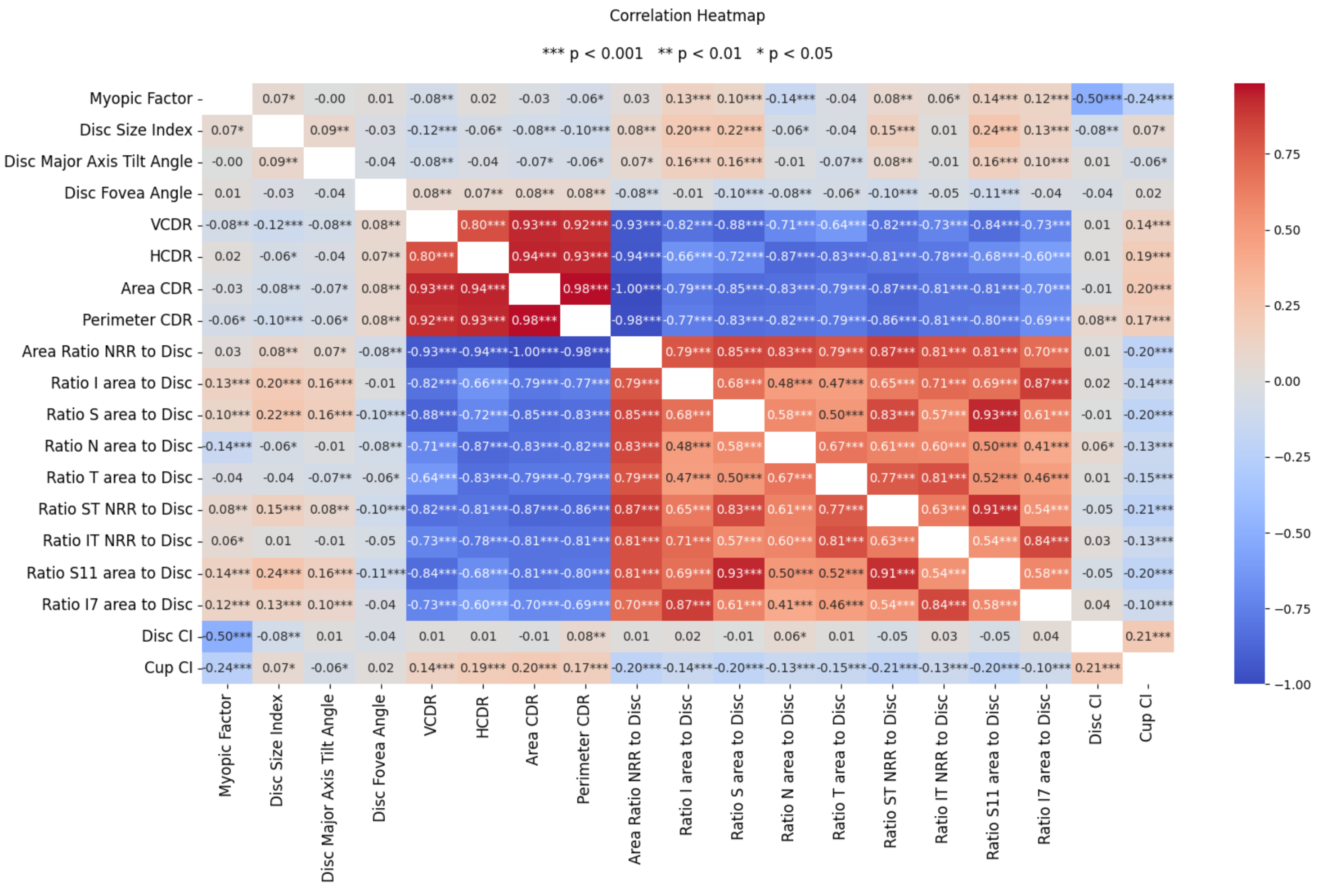

Supplementary Figure 6 | Correlation coefficient heatmap among different optic disc features in Tohoku University dataset of primary open angle glaucoma (POAG) eyes. The correlation heatmap is similar to the AIROGS dataset as discussed in the manuscript.

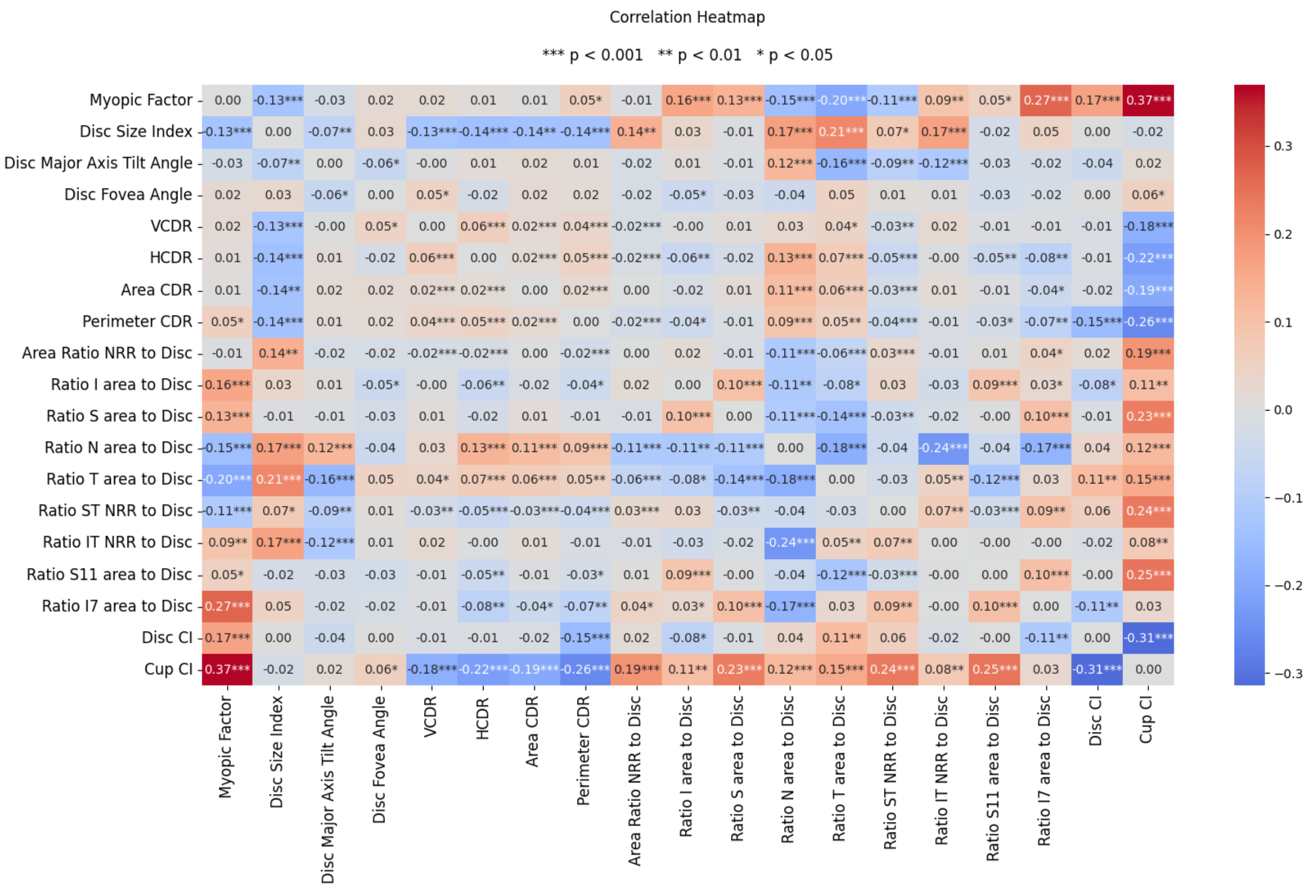

Supplementary Figure 7 | Correlation coefficient heatmap difference of normal and normal tension glaucoma (NTG) analyzed with bootstrap method. Star marks suggest shows the statistical significance (i.e. p value). There are noteworthy variations in the heatmap of correlation coefficient differences among NTG and POAG groups.

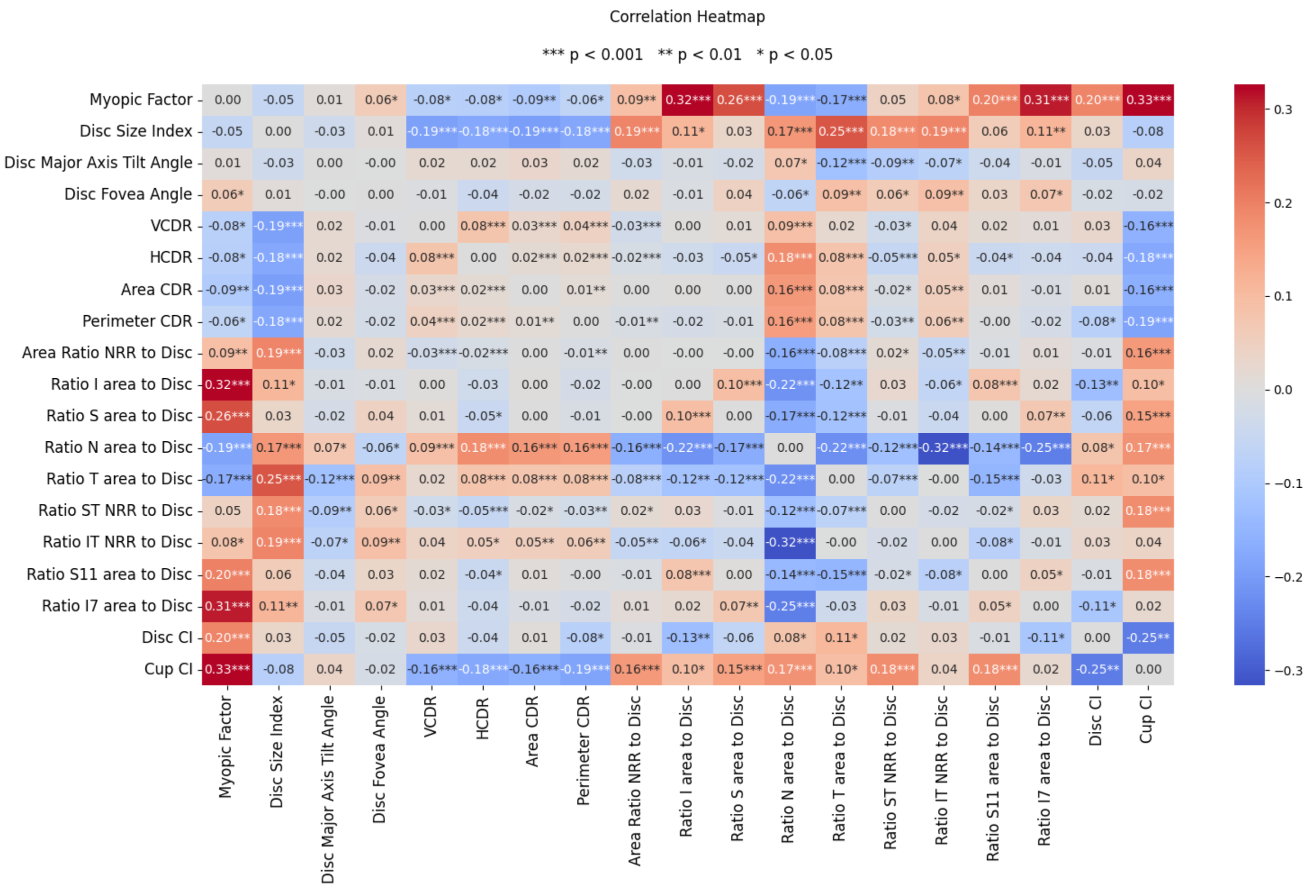

Supplementary Figure 8 | Correlation coefficient heatmap difference of normal and primary open angle glaucoma (POAG) analyzed with bootstrap method. Star marks suggest shows the statistical significance (i.e. p value). It is like that of AIROGS, suggesting prevalence of POAG glaucoma type in AIROGS dataset.

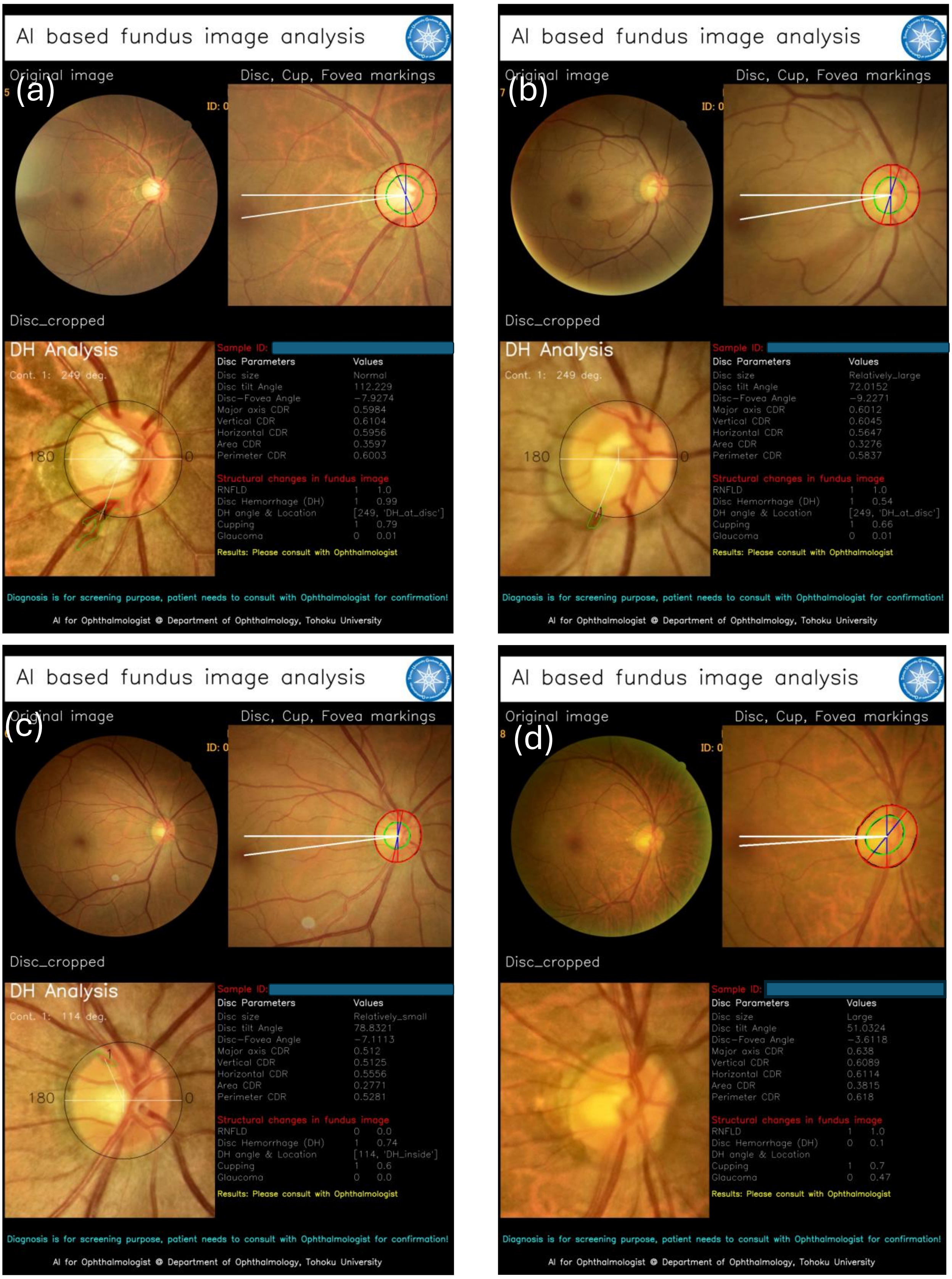

Supplementary Figure 9 | Examples of glaucoma referral and non-referral cases from a real-world screening dataset.

(a)-(c) were predicted as normal by the single DL-based binary classification model, MTL_LWBNA-unet, whereas the screening ophthalmologist flagged them as glaucoma cases. (d) depicts a fundus image marked as normal by both the screening ophthalmologist and the single DL-based model but flagged as glaucoma by the AI-GS network.

In (a), the AI-GS network correctly identified pathological features such as cupping, RNFLD, and DH, classifying it as a glaucoma referral. This decision aligns well with those of glaucoma experts and the screening ophthalmologist.

(b) shows a case with evident RNFLD and slight cupping, where the AI-GS network's decision also aligns with those of glaucoma experts and the screening ophthalmologist.

(c) illustrates a challenging case marked as normal by all three experts but flagged by the AI-GS network as a glaucoma referral due to the detection of DH in the superior temporal region and minor cupping. This highlights the AI-GS network's sensitivity in identifying subtle pathological features that may be overlooked by experts.

(d) was marked as a non-referral case by both the screening ophthalmologist and the single DL-based model, but the AI-GS network flagged it as a glaucoma referral by identifying RNFLD and minor cupping. Upon careful examination by a glaucoma expert, the presence of RNFLD in the inferior-temporal region was confirmed. The location of RNFLD appears to match a slight notching of the cup.

This demonstrates the importance of the AI-GS network in detecting minor pathological features, which can assist in reconsidering positive cases that may have been missed during screening due to time constraints.

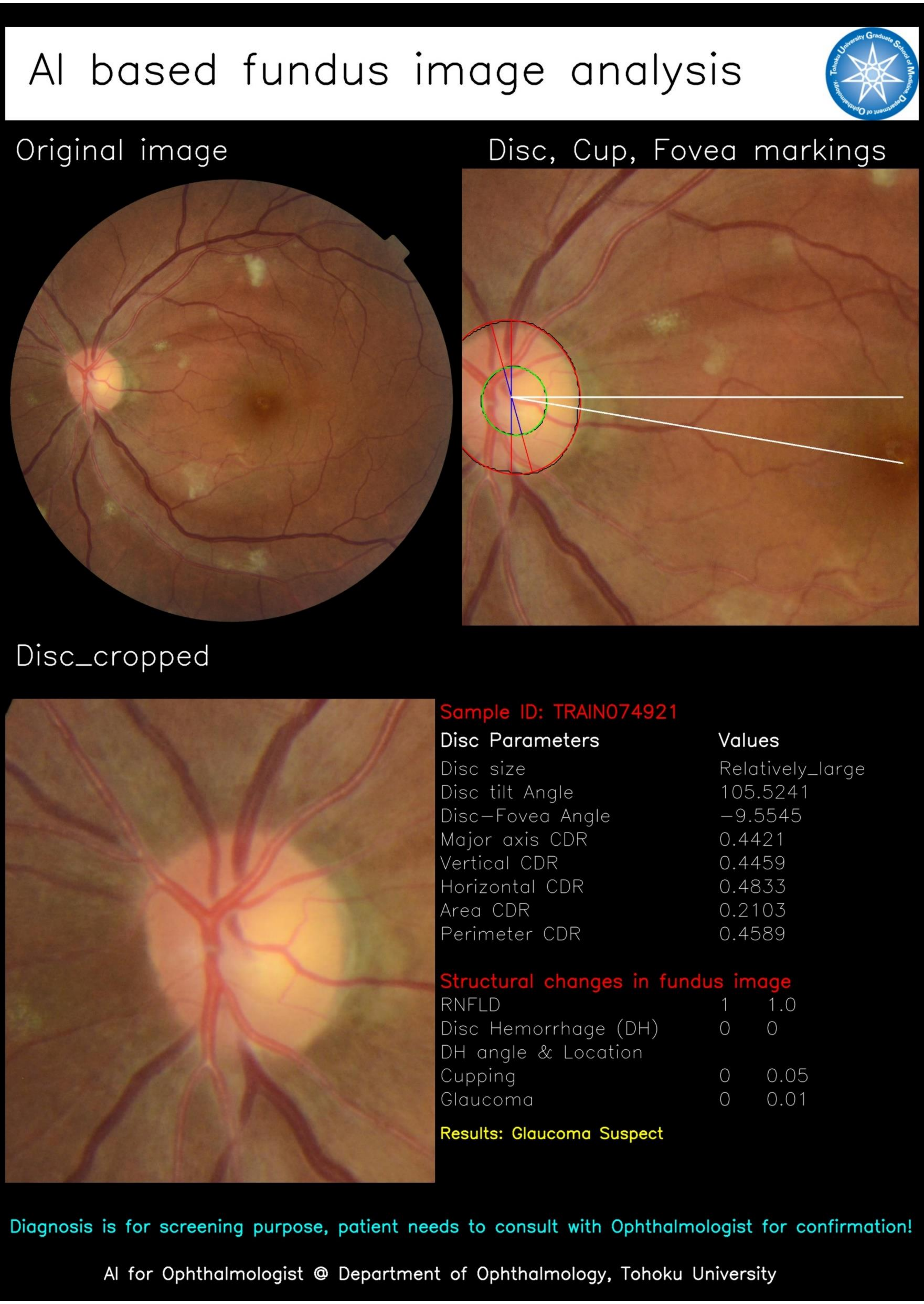

Supplementary **Figure 10 | Diabetic retinopathy example of fundus image from AIROGS dataset (non-referral glaucoma).** AI-GS network detected RNFLD+ but prediction for glaucoma is negative based on DL and cupping-based decisions.

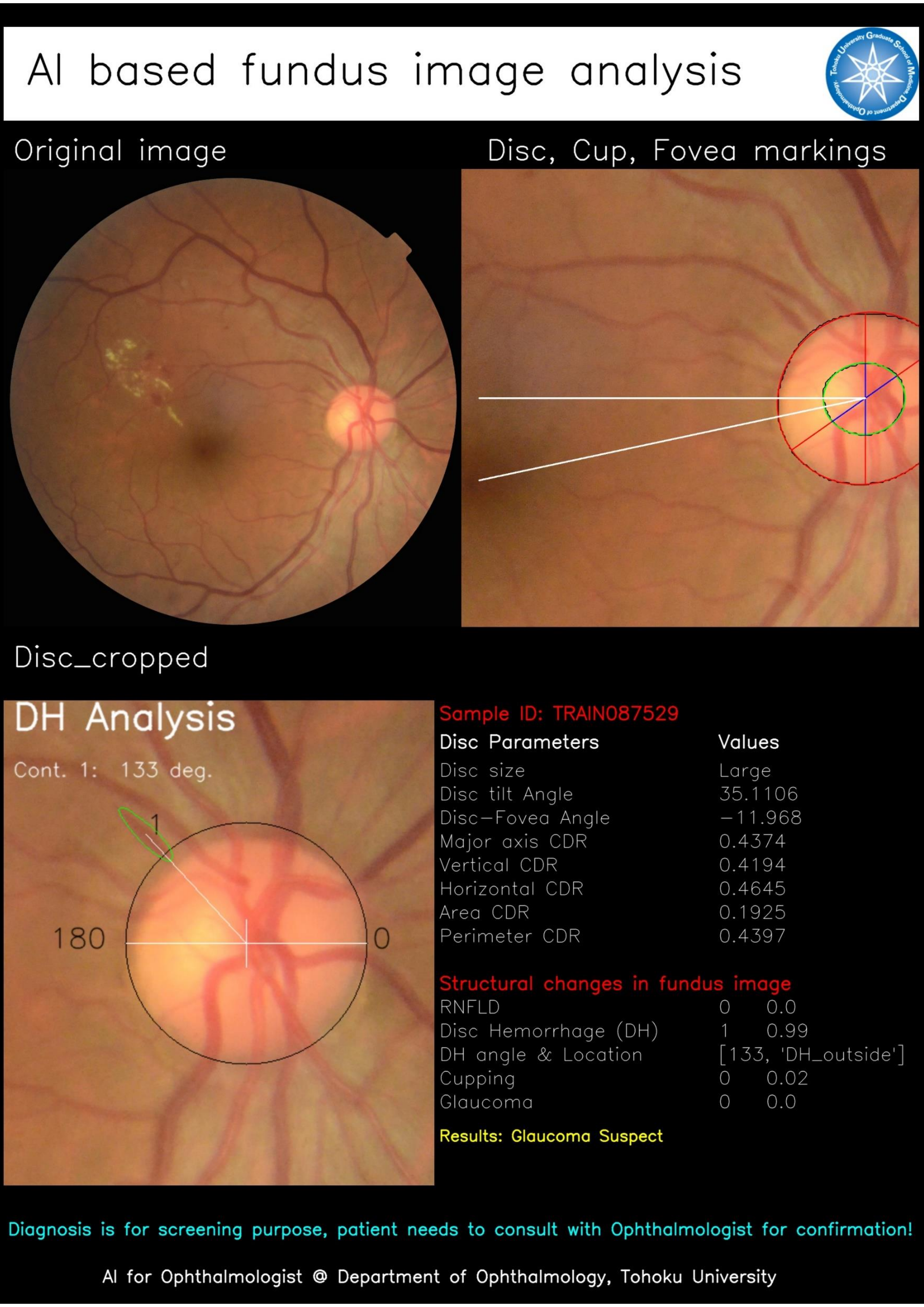

Supplementary **Figure 11 | BRVO Example of fundus image from AIROGS dataset (non-referral glaucoma)**. AI-GS network detected DH+ but prediction for glaucoma is negative based on DL and cupping-based decisions.

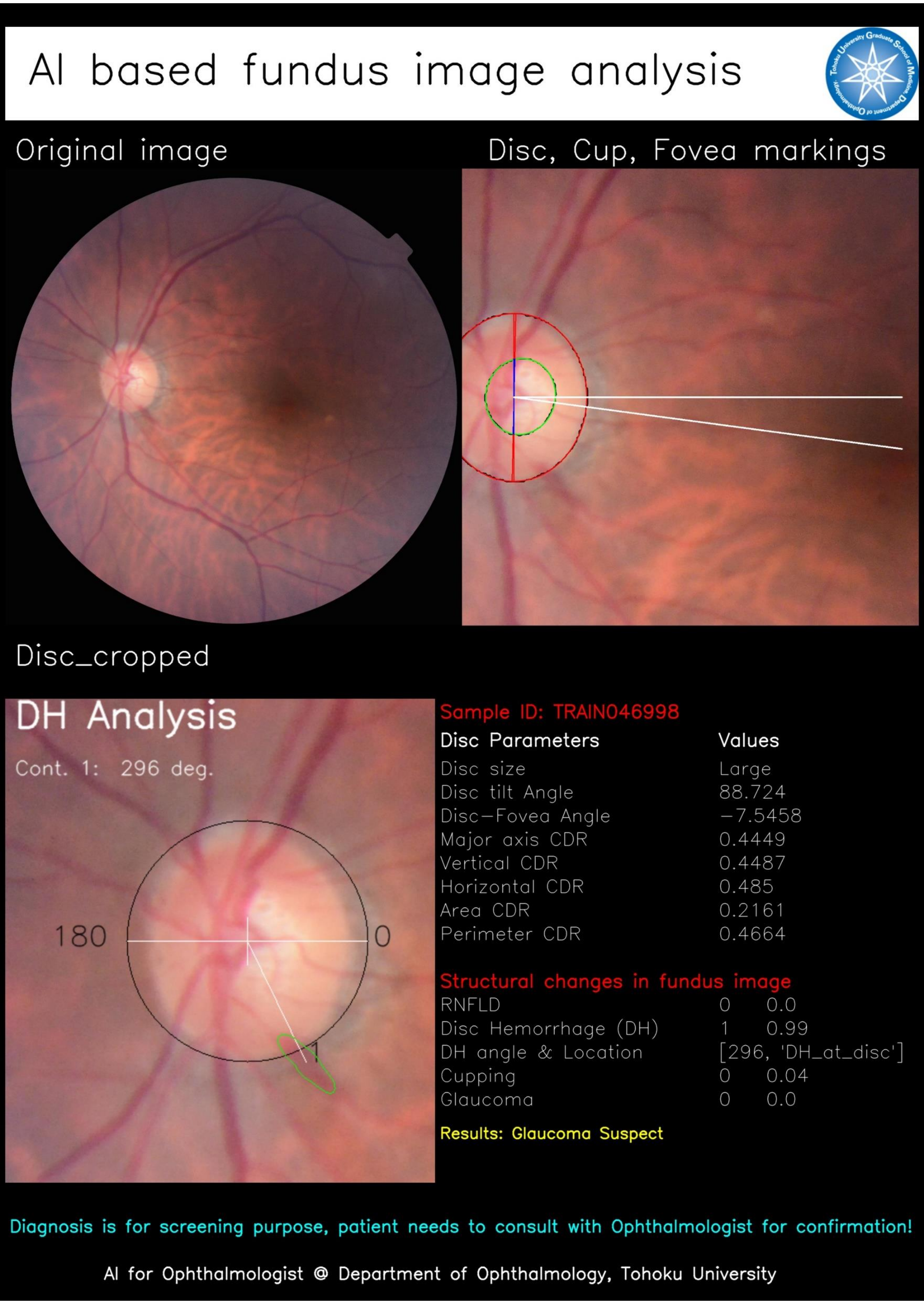

Supplementary Figure 12 | Glaucoma Suspect Example of fundus image from AIROGS dataset (non-referral glaucoma). AI-GS network detected DH+ but prediction for glaucoma is negative based on DL and cupping-based decisions.

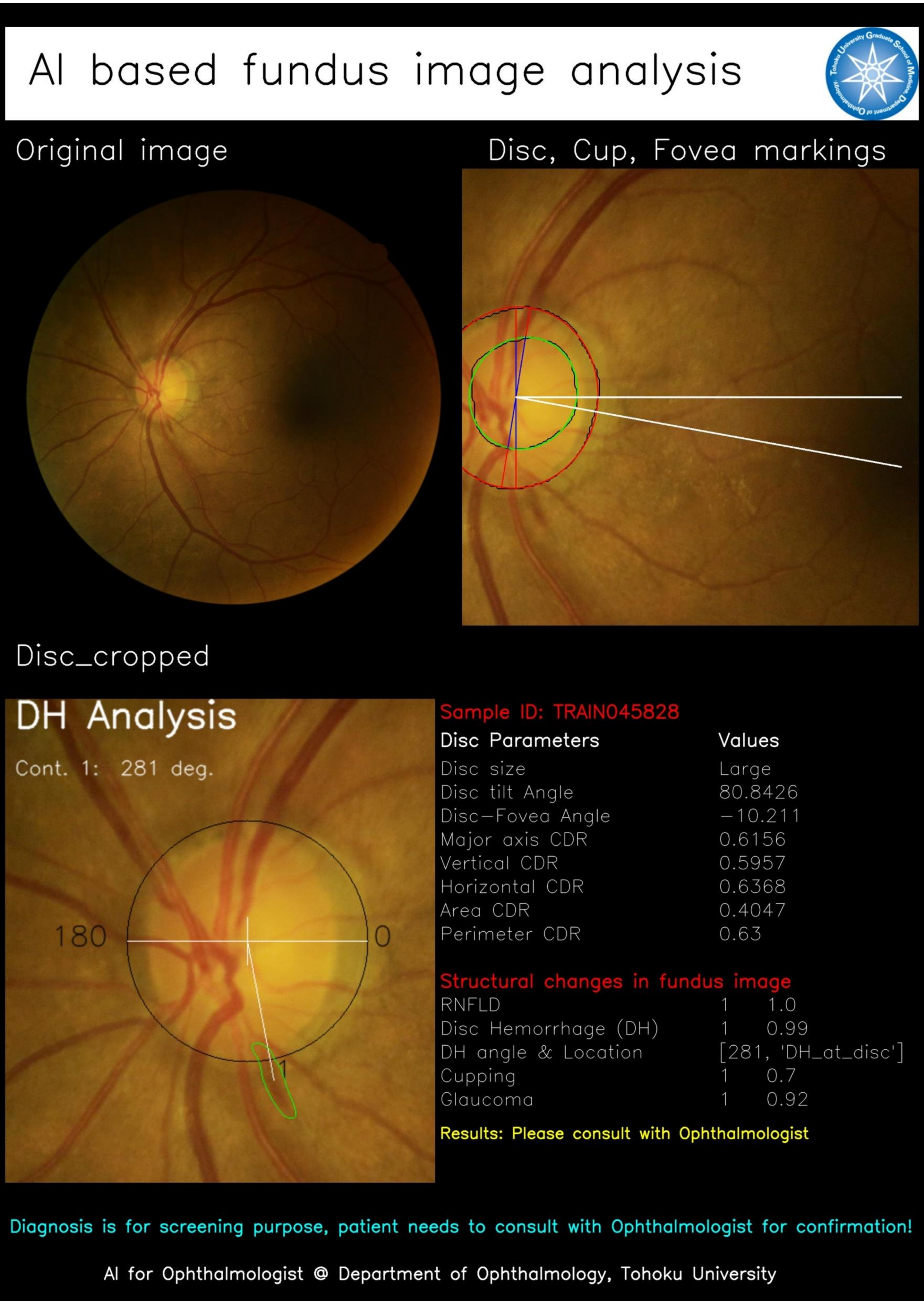

Supplementary **Figure 13 | Example of fundus image from AIROGS dataset (non-referral glaucoma)**. AI-GS network detected all the features related to glaucoma, and it appears to be the case of glaucoma, but without the visual field testing, it can not be confirmed.

Supplementary Table 1| Reanalysis of fundus images flagged as ‘Glaucoma’ by the screening ophthalmologist but predicted as ‘Normal’ by the single binary classification DL model in the Miyagi screening dataset (Table 1 in the paper).

Three glaucoma experts (NT, TN, MS) independently reviewed the images without prior knowledge of AI predictions or screening outcomes. Significant diagnostic variability was observed: the screening ophthalmologist, prioritizing sensitivity to minimize missed diagnoses, flagged all 230 cases as glaucoma. Glaucoma experts diagnosed 206, 178, and 172 cases, reflecting differences in diagnostic criteria, clinical experience, and thresholds. The MTL binary classification model failed entirely, classifying all cases as "Normal," highlighting its poor sensitivity for real-world screening. In contrast, the AI-GS network correctly identified 127 glaucoma cases, while the Adjusted AI-GS network further improved this to 193, closely aligning with expert diagnoses.

Model/Network/Screening/General/Expert Ophthalmologist	Normal	Glaucoma
Screening Ophthalmologist	0	230
MTL_LWBNA-unet (Binary classification)	230	0
AI-GS network	103	127
Adjusted AI-GS network	37	193
Glaucoma Expert-1	52	178
Glaucoma Expert-2	24	206
Glaucoma Expert-3	58	172
Agreement Glaucoma Expert-1 & 2	14	168
Agreement Glaucoma Expert-1 & 3	18	138
Agreement Glaucoma Expert-2 & 3	13	162
Agreement Glaucoma Experts-1, 2, 3	9	132
Agreement All Glaucoma Experts & AI-GS	7	87
Agreement All Glaucoma Experts & Adjusted AI-GS network	4	120
